# Effectiveness of a complex regional advance care planning intervention to improve care consistency with care preferences: study protocol for a multi-center, cluster-randomized controlled trial focusing on nursing home residents (BEVOR trial)

**DOI:** 10.1186/s13063-022-06576-3

**Published:** 2022-09-12

**Authors:** Kornelia Götze, Claudia Bausewein, Berend Feddersen, Angela Fuchs, Amra Hot, Eva Hummers, Andrea Icks, Änne Kirchner, Evelyn Kleinert, Stephanie Klosterhalfen, Henrike Kolbe, Sonja Laag, Henriette Langner, Susanne Lezius, Gabriele Meyer, Joseph Montalbo, Friedemann Nauck, Christine Reisinger, Nicola Rieder, Jan Schildmann, Michaela Schunk, Henrikje Stanze, Christiane Vogel, Karl Wegscheider, Antonia Zapf, Georg Marckmann, Jürgen in der Schmitten, on behalf of the BEVOR study group, Annika Albert, Cornelia Alheid, Claudia Bausewein, Manuela Bruene, Christian Calles, Havva Camci, Anne Daubmann, Sophie Dahlke, Stephanie Enger, Berend Feddersen, Gerd Felder, Carsten Fluck, Andreas Freienstein, Theresa Freytag, Angela Fuchs, Andrea Icks, Jürgen in der Schmitten, Lena Hensel, Eva Hummers, Amra Hot, Änne Kirchner, Evelyn Kleinert, Stephanie Klosterhalfen, Henrike Kolbe, Sonja Laag, Henriette Langner, Susanne Lezius, Georg Marckmann, Gabriele Meyer, Jospeh Montalbo, Friedemann Nauck, Thuy Nguyen, Andre Nowak, Malte Ossenberg, Christine Reisinger, Sophia Reuter, Nicola Rieder, Tanja Riester, Irina Rosu, Holger Rösgen, Katharina Salanta, Zeinep Sassi, Jan Schildmann, Thomas Schulenberg, Michaela Schunk, Daniela Sommer, Henrikje Stanze, Andreas Stöhr, Anke Theuerkauf, Nancy Thilo, Jessica Tönjann, Mahnaz Partowinia-Peters, Sebastian Prommersberger, Susanne Przybylla, Christiane Vogel, Markus Vomhof, Janka Wilken, Antonia Zapf, Jennifer Zimprich

**Affiliations:** 1 Institute of General Practice, Medical Faculty, Heinrich Heine University Düsseldorf, Düsseldorf, Germany; 2 Department of Palliative Medicine, Munich University Hospital, Munich, Germany; 3 Institute of Medical Biometry and Epidemiology, University Medical Center Hamburg-Eppendorf, Hamburg, Germany; 4 Department of General Practice, University Medical Center Göttingen, Göttingen, Germany; 5 Institute for Health Services Research and Health Economics, German Diabetes Center, Leibniz Center for Diabetes Research at the Heinrich Heine University Düsseldorf, Düsseldorf, Germany; 6 Institute of Health and Nursing Science, Medical Faculty, Martin Luther University Halle-Wittenberg, Halle (Saale), Germany; 7 Coordination Center for Clinical Trials – KKSD, Heinrich Heine University Düsseldorf, Düsseldorf, Germany; 8 Barmer Health Insurance, Wuppertal, Germany; 9 Department of Palliative Medicine, University Medical Center Göttingen, Göttingen, Germany; 10 Institute of Ethics, History and Theory of Medicine, Ludwig Maximilians University Munich, Munich, Germany; 11 Institute for History and Ethics of Medicine, Martin Luther University Halle-Wittenberg, Halle (Saale), Germany; 12 Department of Social and Nursing Science, City University of Applied Science Bremen, Bremen, Germany; 13 Institute of Family Medicine/General Practice, Medical Faculty, University of Duisburg-Essen, Essen, Germany

**Keywords:** Advance care planning, Nursing homes, ACP facilitation, Complex intervention, Cluster-randomized controlled trial, Study protocol, Patient-centered care

## Abstract

**Background:**

According to recent legislation, facilitated advance care planning (ACP) for nursing home (NH) residents is covered by German sickness funds. However, the effects of ACP on patient-relevant outcomes have not been studied in Germany yet. This study investigates whether implementing a complex regional ACP intervention improves care consistency with care preferences in NH residents.

**Methods:**

This is a parallel-group cluster-randomized controlled trial (cRCT) with 48 NHs (≈ 3840 resident beds) between 09/2019 and 02/2023. The intervention group will receive a complex, regional ACP intervention aiming at sustainable systems redesign at all levels (individual, institutional, regional). The intervention comprises comprehensive training of ACP facilitators, implementation of reliable ACP processes, organizational development in the NH and other relevant institutions of the regional healthcare system, and education of health professionals caring for the residents. Control group NHs will deliver care as usual.

Primary outcome is the hospitalization rate during the 12-months observation period. Secondary outcomes include the rate of residents whose preferences were known and honored in potentially life-threatening events, hospital days, index treatments like resuscitation and artificial ventilation, advance directives, quality of life, psychological burden on bereaved families, and costs of care.

The NHs will provide anonymous, aggregated data of all their residents on the primary outcome and several secondary outcomes (data collection 1). For residents who have given informed consent, we will evaluate care consistency with care preferences and further secondary outcomes, based on chart reviews and short interviews with residents, surrogates, and carers (data collection 2). Process evaluation will aim to explain barriers and facilitators, economic evaluation the cost implications.

**Discussion:**

This study has the potential for high-quality evidence on the effects of a complex regional ACP intervention on NH residents, their families and surrogates, NH staff, and health care utilization in Germany. It is the first cRCT investigating a comprehensive regional ACP intervention that aims at improving patient-relevant clinical outcomes, addressing and educating multiple institutions and health care providers, besides qualification of ACP facilitators. Thereby, it can generate evidence on the potential of ACP to effectively promote patient-centered care in the vulnerable population of frail and often chronically ill elderly.

**Trial registration:**

ClinicalTrials.gov ID NCT04333303. Registered 30 March 2020.

**Supplementary Information:**

The online version contains supplementary material available at 10.1186/s13063-022-06576-3.

## Administrative information

Note: the numbers in curly brackets in this protocol refer to SPIRIT checklist item numbers. The order of the items has been modified to group similar items (see http://www.equator-network.org/reporting-guidelines/spirit-2013-statement-defining-standard-protocol-items-for-clinical-trials/).

**Table:** 

Title {1}	**Effectiveness of a complex regional advance care planning intervention to improve care consistency with care preferences: study protocol for a multi-center, cluster-randomized controlled trial focusing on nursing home residents (BEVOR trial)**
Trial registration {2a and 2b}	**ClinicalTrials.gov****ID:** NCT04333303 Registration date: 31.03.2020
Protocol version {3}	2022-05
Funding {4}	Innovation Fund of the German Federal Joint Committee *(Gemeinsamer Bundesausschuss),* funding number: 01VSF18004
Author details {5a}	**First and corresponding Author:** **Götze Kornelia** Institute of General Practice, Medical Faculty, Heinrich Heine University Düsseldorf, Germany; Email: goetzeko@uni-duesseldorf.de); **Last Authors (shared last authorship)** **Marckmann Georg** Institute of Ethics, History and Theory of Medicine, Ludwig Maximilians University Munich, Germany; Email: marckmann@lmu.de; **in der Schmitten Jürgen** Institute of Family Medicine/General Practice, Medical Faculty, University of Duisburg-Essen, Germany; Email: jids@uk-essen.de **Further authors** **Fuchs A, Klosterhalfen S,** Institute of General Practice, Medical Faculty, Heinrich Heine University Düsseldorf, Germany; Angela Fuchs (angela.fuchs@med.uni-duesseldorf.de), Stephanie Klosterhalfen (stephanie.klosterhalfen@med.uni-duesseldorf.de); **Reisinger C** Institute of Ethics, History and Theory of Medicine, Ludwig Maximilians University Munich, Germany; **Icks A, Montalbo J** Institute for Health Services Research and Health Economics, German Diabetes Center, Leibniz Center for Diabetes Research at the Heinrich Heine University Düsseldorf, Germany; Andrea Icks (andrea.icks@uni-duesseldorf.de), Joseph Montalbo (joseph.montalbo@ddz.de); **Meyer G, Kirchner Ä, Langner H** Institute of Health and Nursing Science, Medical Faculty, Martin Luther University Halle-Wittenberg, Germany; Gabriele Meyer (gabriele.meyer@medizin.uni-halle.de), Änne Kirchner (aenne.kirchner@medizin.uni-halle.de), Henriette Langner (henriette.langner@medizin.uni-halle.de); **Zapf A, Wegscheider K, Lezius S, Hot A** Institute of Medical Biometry and Epidemiology, University Medical Center Hamburg-Eppendorf, Germany; Antonia Zapf (a.zapf@uke.de), Karl Wegscheider (k.wegscheider@uke.de), Susanne Lezius (s.lezius@uke.de), Amra Hot (a.hot@uke.de); **Kolbe H, Przybylla S** Coordination Center for Clinical Trials – KKSD, Heinrich Heine University Düsseldorf, Germany; Henrike Kolbe (henrike.kolbe@med.uni-duesseldorf.de), Susanne Przybylla (susanne.przybylla@med.uni-duesseldorf.de); **Bausewein C, Feddersen B, Schunk M** Department of Palliative Medicine, Munich University Hospital, Germany; Claudia Bausewein (claudia.bausewein@med.uni-muenchen.de), Berend Feddersen (berend.feddersen@med.uni-muenchen.de), Michaela Schunk (michaela.schunk@med.uni-muenchen.de); **Hummers E, Kleinert E** Department of General Practice, University Medical Center Göttingen, Germany; Eva Hummers (eva.hummers@med.uni-goettingen.de), Evelyn KIeinert (evelyn.kleinert@med.uni-goettingen.de); **Nauck F, Rieder N** Department of Palliative Medicine, University Medical Center Göttingen, Germany; Friedemann Nauck (friedemann.nauck@med.uni-goettingen.de), Nicola Rieder (nicola.rieder@med.uni-goettingen.de); **Stanze H** Department of Social and Nursing Science, City University of Applied Science Bremen, Germany; Henrikje Stanze (Henrikje.Stanze@hs-bremen.de) **Schildmann J, Vogel C** Institute for History and Ethics of Medicine, Martin Luther University Halle-Wittenberg, Germany; Jan Schildmann (jan.schildmann@medizin.uni-halle.de), Christiane Vogel (christiane.vogel@medizin.uni-halle.de); **Laag S** Barmer health insurance, Wuppertal; Germany; Sonja Laag (Sonja.laag@barmer.de); **on behalf of the BEVOR study group**
Name and contact information for the trial sponsor {5b}	**Heinrich Heine University Düsseldorf** Acting on behalf: Düsseldorf University Hospital Represented by CCO Dipl. Kfm. Ekkehard Zimmer Moorenstr. 5 40225 Düsseldorf Germany
Role of sponsor {5c}	The funder was not involved in the design of the study and will not be involved in the collection, management, analysis or interpretation of data, writing of any reports, or the decision to submit any report for publication.

## Introduction

### Background and rationale {6a}

When frail, chronically ill persons lose decision-making capacity, they often receive default life-sustaining treatment even though many, if asked, would not have consented [1]. For example, excess hospitalization rates of nursing home (NH) residents in their final year of life have severe medical, ethical, and economic implications: poor prognosis, severe course of disease, frequent hospital readmissions without informed consent, disregard of autonomous choices, and high costs [2] of unwanted and potentially harmful interventions [3].

The emerging concept of advance care planning (ACP) [4, 5] aims at improving consistency between patients’ care preferences and care delivered in patients who are not capable of decision-making at the time when critical decisions need to be taken [6, 7]. To this end, ACP, in contrast to the conventional approach to advance directives (AD) [8], entails conversations with specifically qualified staff (“ACP facilitators”) that enable persons and/or their surrogates to make well-informed decisions about life-sustaining treatment before a life-threatening health crisis may occur in the future [9]. Besides this conversational process as the core element of ACP on the individual level, successful ACP requires educational efforts and a fundamental systems redesign in institutions and services that are part of these patients’ care in order to ensure that patients’ preferences are reliably known and honored when these patients cannot decide for themselves [10, 11]. Systematic reviews [12–14] indicate that ACP increases completion of ADs [15], improves concordance between preferences for care and delivered care [9], promotes end-of-life care discussions, and reduces decisional conflict [13]. ACP has also been shown to decrease rates of psychological trauma, depression and anxiety in relatives, existential concerns, hospitalizations, life-sustaining interventions like cardiopulmonary resuscitation, and hospital as last place of care [14]. In NHs, ACP has been found to improve routines and culture, documentation of preferences, and adherence to such documents. Interventions have resulted in fewer hospital admissions and hospital deaths and in an increase of NH as place of death, in improved communication and decreased staff distress [12, 16, 17]. Data on cost-effectiveness is scarce, but ACP, if comprehensively understood and successfully implemented, may have the potential to offset program costs by corresponding savings [2].

Some studies have failed to demonstrate positive effects of ACP, and critiques worry that ACP may be overrated [18]. However, there are methodological challenges and pitfalls, and also different conceptions and expectations, impeding an overall judgment of ACP [19]. An ACP trial in Dutch NHs did not reveal an impact on patient activation (PAM-Score) and quality of life (12-Item Short Form Survey (SF-12)), which are, however, no plausible aims of ACP [6, 20]. Similarly, in a large European cluster-randomized trial (cRCT) on hospitalized patients with advanced cancer, quality of life and symptoms did not improve through ACP [21, 22]. Again, these outcomes could not be expected to be relevantly influenced by ACP, given its definition and aims [4, 6]. In addition, both studies’ ACP interventions were confined to the individual (conversational) level although the effect of ACP on patient-centered outcomes is known to depend on implementation and systems redesign as much as on individual facilitation [23].

In Germany, legislation in effect since 2018 has allowed NHs to optionally offer facilitated ACP to their residents [24, 25]. This creates a unique opportunity to study clinical outcomes of ACP programs in German NHs in order to assess whether the significant investment in ACP deems justified by improved patient-centered decision-making. Furthermore, while a German ACP program implemented in 2009–2011 (*beizeiten begleiten*) has proven its efficacy for increasing AD prevalence [1], its effects on patient-relevant outcomes have not been investigated so far. Given that ACP is deemed highly sensitive to culture and healthcare system, many published trials are methodologically weak, and only few RCTs so far evaluated patient-centered outcomes of clinical relevance, there is a need to investigate the effectiveness of ACP in German NHs before it has been broadly adopted in NHs, or is to be extended to other target populations [26]. Combining this with a health economic and process evaluation will enable us to better understand the economic background and to identify barriers and facilitating factors for a wide-scale implementation of ACP in German nursing homes.

### Objectives {7}

To investigate the effectiveness of a comprehensive regional ACP intervention aiming at improved care consistency with care preferences in German NH residents and to identify facilitators and barriers of the implementation process.

The following hypotheses are investigated in the intervention group compared to the control group:

- In potentially life-threatening events, residents’ treatment preferences are more often known and honored;
- Rates of predefined unwanted life-sustaining interventions (cardiopulmonary resuscitation, invasive ventilation, intensive care treatment, tube feeding, and others) are reduced;
- Hospital admissions and hospital days are reduced;
- The perception that in deceased residents, care delivered was consistent with care preferences, is higher among bereaved families and staff, and the psychological burden on families is lower;
- Last place of care is more often in the NH;
- The intervention costs are at least partially offset by consequential cost savings.

### Trial design {8}

**Fig. 1:**
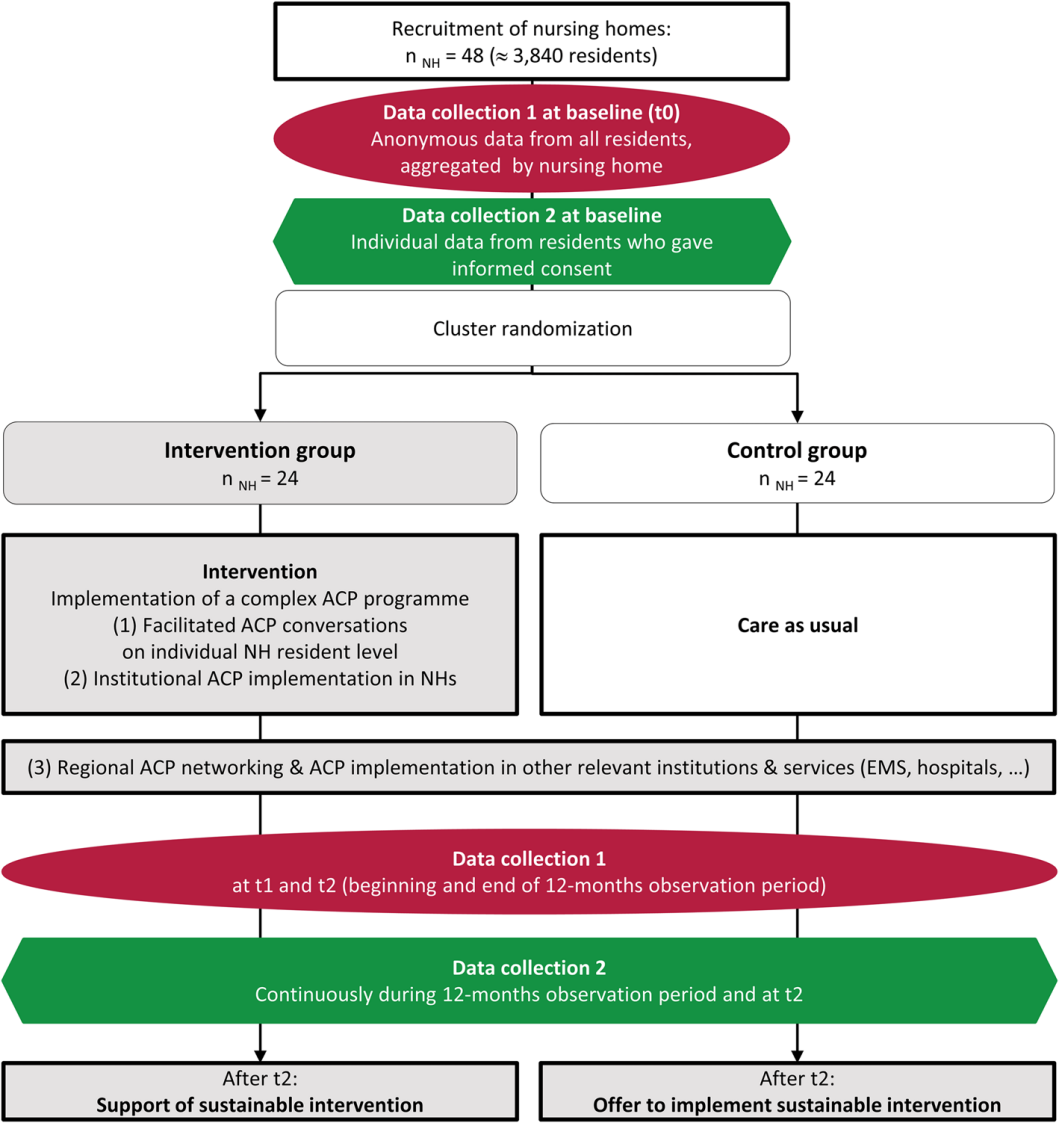
Flow chart of data collections and interventions throughout the trial. *Caption*: *ACP* advance care planning, *EMS* emergency medical service. t0: baseline, t1: end of run-in phase of the intervention / begin of 12-months observation period, t2: end of observation period

The BEVOR study is a multi-center, parallel-group, cluster-randomized controlled trial (cRCT) investigating the effects of a comprehensive ACP intervention focusing on NH residents with embedded process and health economic evaluation. Randomization will be performed at the cluster level. A cluster is defined as a NH and the observation unit as a resident. The intervention group will receive the ACP intervention while the control group continues with care as usual. Figure 1 shows recruitment, randomization, and measurement points.

The health economic evaluation will be conducted alongside the BEVOR trial based on data collection 2. Cost-consequences and cost-effectiveness will be calculated from the perspective of the German social insurance (statutory health insurance and long-term care insurance).

The outcome evaluation of the cluster RCT will be accompanied by a process evaluation. The design of the process evaluation is informed by the UK Medical Research Council (MRC) framework for process evaluations of complex interventions [27]. We will assess context factors, mechanisms of impact, and the implementation process using a mixed methods design, collecting data throughout implementation period of the intervention.

## Methods: participants, interventions, and outcomes

### Study setting {9}

The study will be conducted in German NHs that formally qualify for the recently introduced reimbursement of ACP facilitation by the statutory health insurance [26]. Reimbursement allows employment of one full-time qualified ACP facilitator per 400 residents, i.e., for smaller NHs, corresponding part-time facilitators will be employed. ACP facilitators may either be employed by the NH themselves or by a cooperating regional health care provider [28].

Intervention and data collection are provided by four study centers: Düsseldorf (North-Rhine Westphalia, West Germany), Göttingen (Lower Saxony, North Germany), Halle Saale (Saxony-Anhalt, East Germany), and Munich (Bavaria, South Germany). They represent rural and urban regions, and different religious traditions: catholic (Düsseldorf, Munich), protestant (Göttingen), and undenominational (Halle).

### Eligibility criteria {10}

#### Eligibility criteria for study team for the intervention

Principal investigator, project coordinator, and leaders of the intervention teams of the four study sites are ACP facilitator-trainers certified by the standards of the German Advance Care Planning Society [29]. Certified ACP facilitators (corresponding standard) will perform ACP conversations with the NH residents. Regional ACP coordinators receive their training as part of the study and will continuously gain support from a certified ACP facilitator-trainer (see “Interventions”).

#### Inclusion and exclusion criteria for NH recruitment

*Inclusion criteria:* commitment of NH carrier and/or NH management to participate in the study. This includes readiness

- To support data collection,
- To develop a palliative care concept (if not already in place),
- If allocated to the intervention group: to support the implementation of ACP and to contribute the required time and staff resources,
- If allocated to the control group: to wait with the implementation of ACP until it will be provided after the observation period.

*Exclusion criteria:*

- Specialization of the entire NH (e.g., only residents with persistent vegetative state, or with specific psychiatric diseases, e.g., dementia).
- The NH offers already ACP conversations with trained facilitators on a regular basis.
- The NH already routinely encourages all residents to document in advance their treatment preference regarding hospitalization in case they become critically ill and are incapable of decision-making (with or without facilitation).
- The NH participates in another study that could interfere with and influence the results of the BEVOR trial.

#### Inclusion and exclusion criteria for residents; data collection 1 (aggregated anonymous data)

*Inclusion:* All residents of participating nursing homes

*Exclusion***:** None

#### Inclusion and exclusion criteria for residents; data collection 2 (with informed consent)

*Inclusion*: All long-term residents of the participating nursing homes at the time of baseline data collection, irrespective of their decisional capacity

*Exclusion*: Residents in respite care

### Who will take informed consent? {26a}

#### Data collection 1

Because data collection 1 will be performed anonymously, yielding data of all residents aggregated on NH level, informed consent is not required.

#### Data collection 2

This data collection requires informed consent by the resident or his/her surrogate. In addition, informed consent will be sought from a trusted person with a close relationship to the respective resident, from the surrogate (if differing from trusted person), and from the reference nurse, in order to be interviewed under defined circumstances.

##### Obtaining consent from residents or (if incapable) their surrogates

NH staff obtains consent from residents and surrogates. One NH staff member will be designated and trained to communicate between study team and NH, and to approach residents and surrogates. Accompanied by a recommendation letter of the NH management, the following documents are being circulated on behalf of the study team to all the long-term residents of the NH and their surrogates in order to obtain informed consent: (a) invitation to participate in the BEVOR study by the study team, (b) information about ACP and the BEVOR study: on data collection, benefits, burden, and risk of the participation, (c) information about data protection, (d) informed consent form for participant and study team.

As long as there has been neither consent nor rejection, NH staff is asked to address residents and/or surrogates face-to-face or by telephone, inviting them a second and eventually a third time. Resident recruitment is terminated 4 months after a NH’s initiation or if all residents have consented or rejected. Nursing home staff will receive a limited compensation for their time devoted to the study, independent from the number of residents consenting.

##### Obtaining consent from surrogate and (if different) trusted person of the residents

Surrogates and/or most trusted person, as identified by the resident, may be approached by the resident him-/herself, or alternatively by the study team. The study team will issue two reminders. Recruitment of surrogates and/or trusted persons of recruited residents may be continued until the end of the observation period.

##### Obtaining consent from reference nurse

A trained study team member will ask the reference nurse for informed consent directly prior to an intended interview.

#### Health economic evaluation

Part of the health economic evaluation is covered by the consent for data collection 2 (see above).

Trained study staff of the health economic team will obtain written consent from regional ACP coordinators, facilitator, and the study team of the intervention before asking for personal data.

#### Process evaluation

There is no written consent required for the anonymous questionnaires administered to nursing home staff, residents, and family. We will obtain written consent from NH management and nursing staff from each facility of the intervention group, emergency medical service staff, general practitioners, and other stakeholders from the local health system, ACP facilitators, and ACP coordinators before conducting interviews and focus groups.

### Additional consent provisions for collection and use of participant data and biological specimens {26b}

There are no collection of biological specimens. If ancillary studies are required, they must be authorized by the steering committee of the syndicate partners. If the signed consent does not cover an authorized ancillary study, a signed consent must be obtained from all study participants. The ethical boards of the syndicate members must have authorized this consent form.

## Interventions

### Explanation for the choice of comparators {6b}

We chose usual care as comparator because to date there are no other ACP programs with proven effectiveness established in Germany.

### Intervention description {11a}

The intervention group will receive a multimodal complex ACP intervention on

**Fig. 2:**
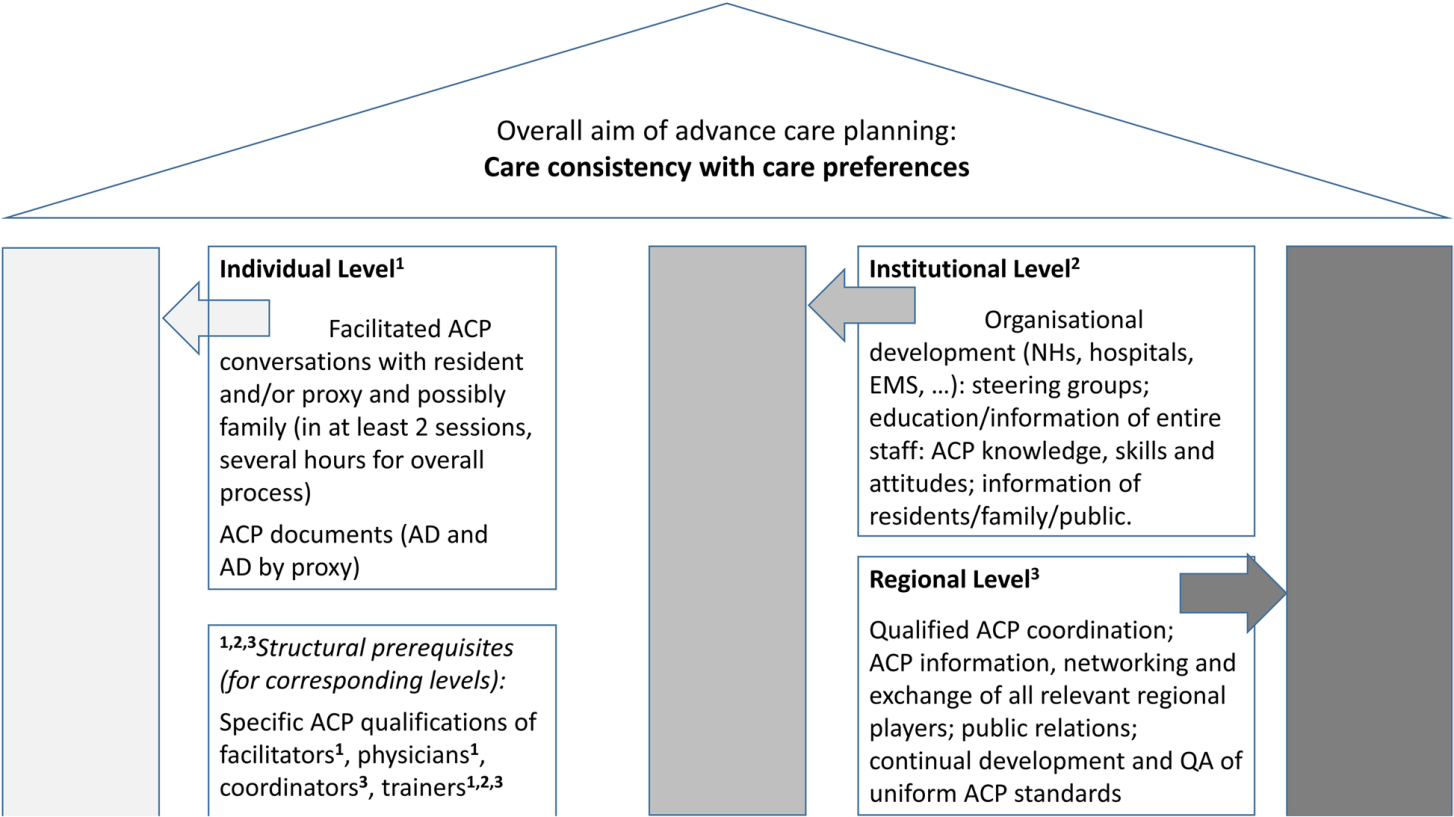
Overview of the elements of the regional ACP program. The intervention elements at the individual level support the residents to plan for future medical care. On the institutional and regional level, a systems redesign and networking is to ensure that the residents’ wishes will be known and honored in case of critical illness accompanied by a lack of decisional capacity. *Caption*: *ACP* advance care planning, *AD *advance directive, *QA* quality assurance. For further information, see additional file 1 (selected intervention details) and 2 (TIDeR Checklist).

- The individual level, i.e., standardized facilitation with NH residents, including where possible their surrogate, family, and their treating physician,
- The institutional level, i.e., organizational redesign of all institutions and services relevant to the healthcare of these NH residents, in particular the participating NHs, the regional hospitals and emergency medical services (EMS), and education of the respective staff, and
- The regional level, i.e., promoting a regional steering group and network of all relevant regional players for regional system redesign (Fig. 2).

The intervention on the individual level is offered to all residents of the participating intervention group NHs, i.e., it is not related to individual resident consent to allow data collection for the BEVOR study.

The intervention on the institutional level, as far as NHs are concerned, is restricted to NHs that belong to the intervention group.

The further interventions on the institutional and regional level are addressed to institutions and services that potentially care for any residents of the NHs participating in the BEVOR study, i.e., both in the intervention and control group, and thus is a potential source of contamination. However, the study’s rationale is that without institutional implementation in the NH, and certainly, without an ACP conversation and its documentation to begin with, contamination of NHs of the control group is unlikely to occur.

The ACP intervention is in accordance with the standards of the German Advance Care Planning Society [29]. Historically, the intervention was derived in 2017 from the former German ACP program *beizeiten begleiten* which was adapted in 2008 from the U.S. program *Respecting Choices* [30], successfully piloted in a scientific project [15], and further developed since in close collaboration with a Swiss ACP project group [31].

#### Individual level

NH residents or, if incapable of decision-making, their surrogates will be offered a structured ACP conversation process with a certified ACP facilitator to reflect on their preferences for future medical care, and if so desired to document them. This conversation process has been shown to last 2 to 3 h (median) over a series of by definition at least two conversations, wherever possible involving designated surrogates and/or family and NH staff if the resident/surrogate so wishes. Treating physicians (mostly general practitioners) will be offered qualification and will be involved in the process, typically at its end. By signing the forms, they explicitly confirm both decisional capacity of the resident, and the resident’s/surrogate’s understanding of implications and consequences of the choices made, thus documenting validity of the AD.

Documentation of the ACP facilitation process using standardized forms for advance directives (AD) or advance directives by proxy (AD-P) follows the standards of the German Advance Care Planning Society [29]. This documentation reflects separate sections of the standardized ACP conversation, framed by the “attitudes towards living, severe illness and death” where the person’s treatment goals and values in case of life-threatening illness are elicited in a deliberative communication process. A written condensation of the “attitudes” forms the AD’s base for documenting concrete treatment preferences in three distinct clinical scenarios: (1) treatment preferences in an emergency (documented on a Physician’s Order for Life-Sustaining Treatment in Case of Emergency (POLST-E)), (2) treatment preferences for hospital care in case of an incapability to consent of uncertain duration, and (3) treatment preferences for a possible future permanent incapability to consent. In surrogate decision-making, besides the “attitudes” two concrete scenarios are documented in the AD-P form: (1) emergency preferences as in the AD, and (2) treatment preferences in case of future severe functional or emotional deterioration [32–34] see Additional file 1 (selected intervention details).

##### Structural prerequisites for these conversations

1. *Non-physician facilitators* are trained to conduct supportive, standardized ACP conversations with NH residents or, if these are incapable of decision-making, with their surrogates. Training by the standards of the German Advance Care Planning Society comprises at least 108 lessons (one lesson = 45 min), including three 3-day workshops, plus, between the workshops, twelve training-facilitations including on-site and telephone coaching (36 lessons). Central component of the workshops are small-group trainings of standardized conversational challenges with simulated patients (24 lessons). For certification, the training must be successfully passed, and many trainees receive extensive additional supervision in order to meet criteria for success. This training also meets the legal standards set by German legislation (executive agreement for § 132g German Social Code V), warranting reimbursement by the statutory health insurance. Facilitator training is delivered by certified ACP facilitator-trainers who, again according to the standards of the German Advance Care Planning Society, are trained as ACP facilitators and participated successfully in a specific ACP trainer workshop with 64 lessons blended learning and over 190 lessons of practical teaching experience as a supervised co-trainer before and after the trainer workshop [35].
2. *Treating physicians* will be offered workshops to understand the ACP process, to support, supervise, and cooperate with facilitators, and to honor advance decisions following the ACP process (4 × 8 lessons = 32 lessons). Program certification will be after the first eight lessons; continuation is recommended.

#### Institutional level

##### Nursing homes

The NHs will receive qualified support for an organizational development contributing to the necessary cultural change, and various degrees of education for all their staff, aiming to ensure that the residents’ treatment preferences are known and honored reliably, and updated when necessary.

1. *An institutional steering group* of the NH is recommended to meet in year 1 about ten times for 1 to 2 h, and afterwards quarterly for sustained program effect. The steering group aims at ensuring organizational redesign, and (later on) sustainability, so that processes support knowing and honoring residents’ preferences. Moderation of the steering group and educational inputs by a qualified study team member is offered.
2. *The entire NH staff* is addressed and informed initially by the ACP trainer (two lessons). It is recommended to repeat this information yearly for sustainability.
3. In the course of year 1, *all nursing and social service staff* are offered eight educational sessions (of one to three lessons each) by the ACP trainer, continually developing knowledge, skills, and attitudes with relevance to ACP, and increasingly building on challenges and questions arising from experiences with the program. Also, the ACP facilitator involves the respective caregiver team in every single finalized ACP process and resulting documentation so that understanding of the program is additionally fostered in a regular routine.
4. *Residents, their relatives, and/or surrogates* will be regularly informed about ACP. Options include newsletters, brochures, flyers, posters, meetings (online or in presence), and a personal approach.

##### Other regional institutions and services

Principally all regional healthcare providers involved in the care of the intervention NH residents will be offered educational training and support to ensure that the residents’ preferences, documented in advance care plans will be known and honored appropriately also at the transferal interfaces, and outside the NH. Qualified study team members and, where available, a regional ACP coordinator will support this process, aiming at pertinent standard procedures for each institution and service.

In particular, emergency medical service teams, hospital staff, and professional guardians will be offered specifically tailored educational packages of two to eight ACP lessons per year. Palliative care and hospice providers, local administration, medical associations, guardianship court, relevant others, and the public are to be yearly informed about the program.

#### Regional level

Beyond individual facilitation and institutional implementation, ACP is to become a regional health policy project if uniform standards of quality and documentation, and sustainability of the regional ACP program, are to be achieved. To this aim, where possible creating a regional ACP network is to be initiated, beginning with the foundation of a regional ACP steering committee. This can become the basis for expanding the program beyond the scope of this study to other target groups in the community, and to install a process of continual quality assurance and development.

The *ACP coordinator*, a new role in the regional healthcare system, is the designated manager of the regional ACP project. So far, there is no regular funding for regional ACP coordinators, but some German communities have granted funding for pilot projects. During the BEVOR study, trainees in this position, including study team members, receive 30 lessons with theoretical and practical inputs and exchange. To a certain extent, study team members will function as regional ACP coordinators, while regional solutions to create a sustainable ACP coordinator workforce beyond this study will be sought and encouraged. ACP coordinators are to engage regional stakeholders. To this end, communication plans, conversations guides, a timeline for further networking steps, and individual expert advice will be provided.

### Criteria for discontinuing or modifying allocated interventions {11b}

Residents in the intervention group are free to accept the offer of an ACP conversation. In addition, the NH managers can discontinue participation in the study at any time. Besides, the intervention package can be tailored to the needs, capacity, or willingness of individual NHs or other institutions. For example, the anticipated frequency or duration of inputs for NH steering groups or staff meetings can be decreased if staff capacity is limited.

### Strategies to improve adherence to interventions {11c}

For adherence to the intervention on the individual level, there will be a close supervision of ACP facilitation by ACP trainers. On the institutional and regional level, study team members seek regular exchange with NH and other staff to solve evolving barriers and problems, and discuss their experiences in at least monthly rounds between the four study centers for calibration. Deviations from the prepared intervention packages will be recorded at each study center in a standardized documentation.

### Relevant concomitant care permitted or prohibited during the trial {11d}

To be able to honor care preferences in life-threatening situations, facilities will be encouraged to establish good emergency and palliative care management. Introducing other ACP programs of any kind during the study period is prohibited.

### Provisions for post-trial care {30}

The intervention aims at implementing a sustainable ACP program that will continue beyond the study. As trial participation does not involve any specific risks, there are no provisions for compensation.

### Outcomes {12}

#### Choice of the primary outcome

The overall goal of ACP is to improve the consistency of care delivered with care preferences [6]. However, directly measuring this consistency in a controlled trial is methodologically challenging [36–38]. First, in the control group of a trial (without ACP), due to low prevalence and even lower relevance and validity of ADs, true care preferences related to typical life-threatening clinical scenarios will not be known in most cases, and thus cannot be compared between groups to the care delivered [39]. Second, evaluating care consistency in German NH requires informed consent to full access to sensitive personal data. Achieving the high rates of consent necessary for robust evidence proves increasingly unsuccessful in the NH setting [1], significantly compromising the representativeness of such studies.

We therefore use a surrogate parameter as primary outcome in our study, i.e., the hospital admission rate. If aggregated by NH, it can be collected anonymously for the entire resident population of all participating NHs from electronic administrative data, thus yielding robust, representative evidence. Hospitalization rates of NH residents are with almost 50% exceedingly high in Germany [40]; prior data show that ACP may reduce the rate of non-beneficial and/or unwanted hospitalizations, which are a major concern of many nursing home residents and have significant implications on health care costs and resource utilization [16]. However, it must be borne in mind that lowering hospitalization rates is not a goal of ACP in itself; rather, it is empirically known that many frail elderly people choose their home as their preferred place of death [41]. Thus, hospitalization may count as an acceptable surrogate parameter for care consistency; at the same time, it should be viewed together with data on care preferences.

We will therefore conduct two separate data collections (see Table 1). While the primary outcome hospital admission rate as well as a few secondary outcomes are collected anonymously for *all* residents of the participating NHs (data collection 1), care consistency with care preferences will be assessed in depth in residents who have given informed consent to study participation (data collection 2). Thus, we will obtain robust data on the effectiveness of the ACP intervention based on the surrogate endpoint of reduced hospital admissions while at the same time we will assess in a subset of residents whether any observed change in the primary outcome is in line with the overall aim of the ACP intervention: improved care consistency with care preferences. Primary outcome is the hospitalization rate per 100 residents per year (from t1 to t2) and is measured at the care facility level.

**Table 1:** Outcomes of the BEVOR trial grouped by data sources, and structural data of participating NHs. *Caption:* *3CP* Care consistency with care preferences, *DPOA* durable power of attorney, *EMS* emergency medical services, *Y/COB* year and country of birth, *NH* nursing home, *No.* number of items, *R (D/O)* relevance (domain/overall), according to Sudore et al. [6]

**Structural data of participating NHs; cross-sectional and retrospective survey; source: NH records**						
		**Parameter**	**No.**	**item example(s)**		
		residents	55	no. of residents in past 12 months /on day of data collection, respite vs longterm care)		
		nursing staff		no. of registered/assistent/hired nurses; no. of full equivalents; no. of palliative care experts; total sick leave days per year		
		palliative care		palliative care concept in place; active collaboration with palliative care services		
		hospice care		collaboration with hospice care services		
		ethics consultations		established; number in past 12 months		
		general practioner		no. of general practioners		
		co-payment		defined private co-payment for residents		
**Data Collection 1 (DC-1): Anonymous data of all residents, aggregated on nursing home level; collected at baseline (t0), end of intervention run-in period (t1) and at the end of observation period (t2)**						
**DC-1a: Clinical and ACP data of participating NHs instrument; cross-sectional and retrospective survey; sources: NH records**						
**Domain**	**Subdomain(s)**	**Outcome**	**R (D)**	**R (O)**	**No.**	**item example(s)**
Health Care Outcomes	care utilisation constructs	hospitalisation	1	17	2	rates; total hospital days
		cardiopulmonary resucitation	11	38	2	attempted; discharged alive
		feeding tube	11	38	2	in place; in use
		deaths	5	21	2	number; place of death
Action Outcomes	communication and documentation/ patient and surrogate	surrogate decision maker: decision and documentation	1;2	2;3	2	documented; type (guardian vs. DPOA)
		advance directive (by proxy)	6;7	9;10	4	documented; last update; state-ment regarding hospitalisation; signature of resident
		advance order for emergency treatment	4	7	3	documented; last update; signature of resident
	Communication and documentation/ values and preferen-ces constructs	surrogate agrees to take on role	3	12	1	surrogate's signature documented
		medical records contain ad-vance care plan	7	10	2	Adance directive (AD) or AD by proxy in medical record
		medical record contains physician treatment orders	4	7	1	Any POLST equivalent according to given definition
**Data Collection 2 (DC-2): Individual data of residents who have given informed consent; collected at baseline and during observation period (t1-t2).**						
**DC-2a: Cross-sectional and retrospective survey; sources: NH records, GP records, hospital records, EMS records**						
**Domain**	**Subdomain(s)**	**Outcome**	**R (D)**	**R (O)**	**No.**	**item example(s)**
n.a.		sociodemographic data; after death data			14; 8	Gender, Y/COB, migration, professional degree, move-in date, days since moving in at start of survey; deceased, date of death, insurance, marital status, religion, care level
Health Care Outcomes	care utilisation constructs	hospitalisation	1	17	3	number; total hospital days
		visits of hospital outpatient services			1	number
		cardiopulmomary resucitation			2	attempted
		intensive care unit	4	21	1	days
		artificial ventilation			1	hours
		feeding tube (PEG)			2	insertion; in place
		deaths	5	21	2	number; place of death
		EMS transports			1	number; kind of transport; emergency physician
		general practioner visits (NH)			1	number
		referrals to specialists			1	number (by specialty)
		medication			5	antibiotics/opioids
		palliative care services delivered	9	29	2	no. (general/specialised)
	health status and mental health	quality of life (resident capable of self-rating)	4	53	24	WHO-QoL-old German Version (Winkler et al. 2016)
		quality of life (resident uncapable of self-rating)	n.a.	n.a.	37	Qualidem (Hüsken et al. 2016)
**Domain**	**Subdomain(s)**	**Outcome**	**R (D)**	**R (O)**	**No.**	**item example(s)**
Action Outcomes	communication & documentation/ surrogate	surrogate decision maker	1;2	2;3	2	documented; type (guardian vs. DPOA)
	communication & documentation/ values&preferences	advance directive	6;7	9;10	15	date; form; publisher; content/preferences if stated for specific given scenarios
		advance order for emergency treatment	4	7	15	date; form; publisher; content/preferences if stated for specific given scenarios
		advance directive by proxy	6;7	9;10	9	documented; formal details; content/preferences if stated for specific given scenarios
		surrogate agrees to take on role	3	12	1	surrogate's signature documented
		discuss values and care preferences with clinicians	5	8	1	elicited in chart review/ in interviews after potentially life-threatening events
		discuss values and care preferences with surrogate and/or family&friends	9	35	1	surrogate's and/or family's /friends' signature documented
		medical records contain ad-vance care plan	7	10	2	Adance directive (AD) or AD by proxy in medical record
		medical record contains physician treatment orders	4	7	1	Any POLST equivalent according to given definition
**DC-2b: 3CP; retrospective evaluation of available sources, max. 3 months after potentially life-threatening events identified from NH charts; sources: NH records, interviews with resident/surrogate, family and reference nurse.**						
**Domain**	**Subdomain(s)**	**Outcome**	**R (D)**	**R (O)**	**No.**	**item example(s)**
Quality of Care Outcomes	care consistent with goal constructs/ Care received is consistent w. goals	care consistency with care preferences re. treatment decisions in the face of potentially life-threatening events in the last 3mths	1	1	9	1. Preference known? IF YES:2. Preferene well-informed (i.e., 4 SDM criteria fulfilled)? 3. Preference honored?
**DC-2c: In case of deceased residents, interviews with bereaved family members and reference nurses**						
**Domain**	**Subdomain(s)**	**Outcome**	**R (D)**	**R (O)**	**No.**	**item example(s)**
Quality of Care Outcomes	care consistent with goal constructs/Care received is consistent with goals	Care consistency with care preferences	1	1	> 100	Selected domains of ADBI (Teno et al. 2001): Inform and promote shared decision making (problem score #2), Encourage advance care planning (problem score #3) and Overall Rating Scale for patient focused, family centered care
	satisfaction with care and communication	Surrogate's/family's ratings of quality of death and dying	1	15		
Health Care Outcomes	depression	depression on the side of bereaved family member	1	31	22	IES-R (Maercker et al. 1998)
		(psychological) trauma on the side of bereaved family members	(1)	(31)	14	HADS-d (Herrmann-Lingen et al. 2018)
**Data Collection 3: Health care expenditures**						
**Domain**	**Subdomain(s)**	**Outcome**	**R (D)**	**R (O)**	**No.**	**item example(s)**
Health Care Outcomes	overall health care expenditures		6	25		complex calculation employing parameters from above, and separate assessments of time resources by skilled staff spent to promote the intervention

Data Collection 4: Process evaluation see "Additional file 1"

#### Secondary outcomes

Secondary outcomes selected for this study are categorized below according to the recommendation of a Delphi panel, grouped by domains and subdomains [6]. Compare Table 1. for a listing of the same outcomes, grouped by data sources / modes, and time points of data access, and linked with their respective relevance according to the Delphi panel’s classification.

##### Health Care Outcomes domain

Subdomains: (1) care utilization constructs: several hospitalization parameters (including the primary outcome), cardiopulmonary resuscitation attempts, feeding tube, intensive care, artificial ventilation, EMS transports, last place of care, general practitioner calls at the NH, referrals to specialists, hospice and palliative care service utilization; (2) health status and mental status: self-and proxy-rated quality of life, depression and trauma of bereaved families

##### Action Outcomes domain

Subdomains: (1) communication and documentation: surrogate constructs: surrogate decision-maker; (2) communication and documentation: values and preferences constructs: medical records contain advance care plan (advance directives, advance order for emergency treatment (e.g., POLST), advance directive by proxy), patient decides on a surrogate, document the surrogate decision-maker, surrogate agrees to take on role (signature), discuss values and care preferences with clinicians/family and friends/the surrogate, medical record contains physician treatment orders (emergency POLST).

##### Quality of Care Outcomes domain

Subdomain: (1) care consistent with goal constructs: care received is consistent with care preferences (see separate text section below); (2) satisfaction with care and communication: bereaved surrogates’/families’ ratings of quality of death and dying

#### Structural data and sociodemographics

Structural data are retrieved from the participating nursing homes at three dates at t0, t1, and t2, regarding nursing staff, palliative and hospice care, ethics consultations, and co-payments. Sociodemographic data describe NH residents who gave informed consent. Both data sets are included in Table 1.

#### Measures / instruments

In the following section measuring of care consistency with care preferences, health economics and process evaluation are discussed in detail; all other measures and instruments for the other outcomes are listed in Table 1.

##### *C*are *c*onsistency with *c*are preferences (3CP)

The various measurements used so far in ACP studies seemed not to warrant a sufficiently valid assessment of 3CP in our controlled trial. Many studies limited this assessment on a retrospective evaluation of end-of-life care of deceased persons, to documented care preferences or to hypothetical consistency. However, also when patients have *survived* life-threatening health crises, it is of particular interest to assess whether the treatment delivered was consistent with their preferences. Therefore, we use two different approaches:

1. To capture the extent of 3CP in life-threatening events, we developed a new tool to measure 3CP in the NH setting. Our perspective is to measure in retrospect whether care that was actually delivered in life-threatening situations was consistent with what the resident wanted in that situation. For this purpose, specifically trained data collectors will identify *potentially life-threatening events* (according to a predefined list) and corresponding critical treatment decisions during the last 3 months by asking the reference nurses of all study participants and screening participating residents’ NH records, in particular nursing reports, medication lists, and hospital discharge letters. 3CP will then be reconstructed for every critical treatment decision identified this way, based on relevant record entries and on interviews with the resident (if capable), the surrogate, and the reference nurse. The final assessment whether the care delivered in a potentially life-threatening event was, in retrospect, consistent with the resident’s declared or presumed treatment preferences at the time of decision will be based on an integrated judgment over all available sources and is subdivided into two steps. The first step leads to identifying consistency of the treatment with the reconstructed care preferences in the sense of a consent or dissent, regardless of the quality of the underlying decisional process. In the second step, we will assess whether the resident’s treatment preferences were developed in a process supported by four central elements of shared decision-making (SDM) [42]:
  - ... was supported to understand and appreciate the clinical situation,
  - ..was informed about available treatment options with benefits and risks,
  - ...was given opportunity to deliberate on the available treatment options, and
  -  ...was encouraged to decide between the available options based on the resident's preferences.
2. In order to measure 3CP in end-of-life care as well, we translated and adapted the domains *Inform and promote shared decision-making* (problem score #2) and *encourage advance care planning* (problem score #3), and the *Overall Rating Scale for patient focused, family centered care* of the After Death Bereaved Family Member Interview (ADBI) [43]. We also developed a version of the overall rating scales of the ADBI for validation of the nurse perspective on 3CP. The validity of both instruments will be assessed using data of the BEVOR study in a separate study.

##### Health economic evaluation

Standardized instruments by Chernyak, Ernsting, and Icks [44] and Seidl et al. [45] will be used to record the consumption of healthcare services, i.e., outpatient physician visits, treatment by therapists, hospital admissions, emergency admissions, emergency medical service transportation, pharmaceutical intake, palliative care, hospice care, and care in NHs. Intervention costs will be extracted from an electronic documentation tool and cover time expenses of players to implement the ACP intervention on all three levels.

##### Process evaluation

According to the UK Medical Research Council [46], a concomitant process evaluation aims to analyze the acceptance by and impact of the intervention on residents, their relatives, nursing staff, NH management, and general practitioners. This will identify potential barriers, burden, and facilitating factors.

Key indicators for an effective implementation process cover acceptability, adoption, appropriateness, feasibility, fidelity, penetration, and sustainability [47]. These are to be structured and evaluated by means of core constructs of the Normalization Process Theory [48], particularly at the level of the institutions and the regional health care system. A mixed methods approach comprising (semi-) standardized questionnaires, focus groups, and guideline-supported individual interviews as well as written progress documentation (logbook) will be used for data collection. General conditions of the four individual study regions and in the care facilities will be described as context factors. Furthermore, the research process and the course of the project will be documented.

The process evaluation addresses multiple participants, e.g., NH residents, their surrogate decision-makers or next of kin/ relatives, nurses and management staff of the NH, ACP facilitators and ACP coordinators, general practitioners, and emergency medical services staff. Additional file 4 gives an overview of target population, purpose, and content of the instruments used.

### Participant timeline {13}

**Fig. 3:**
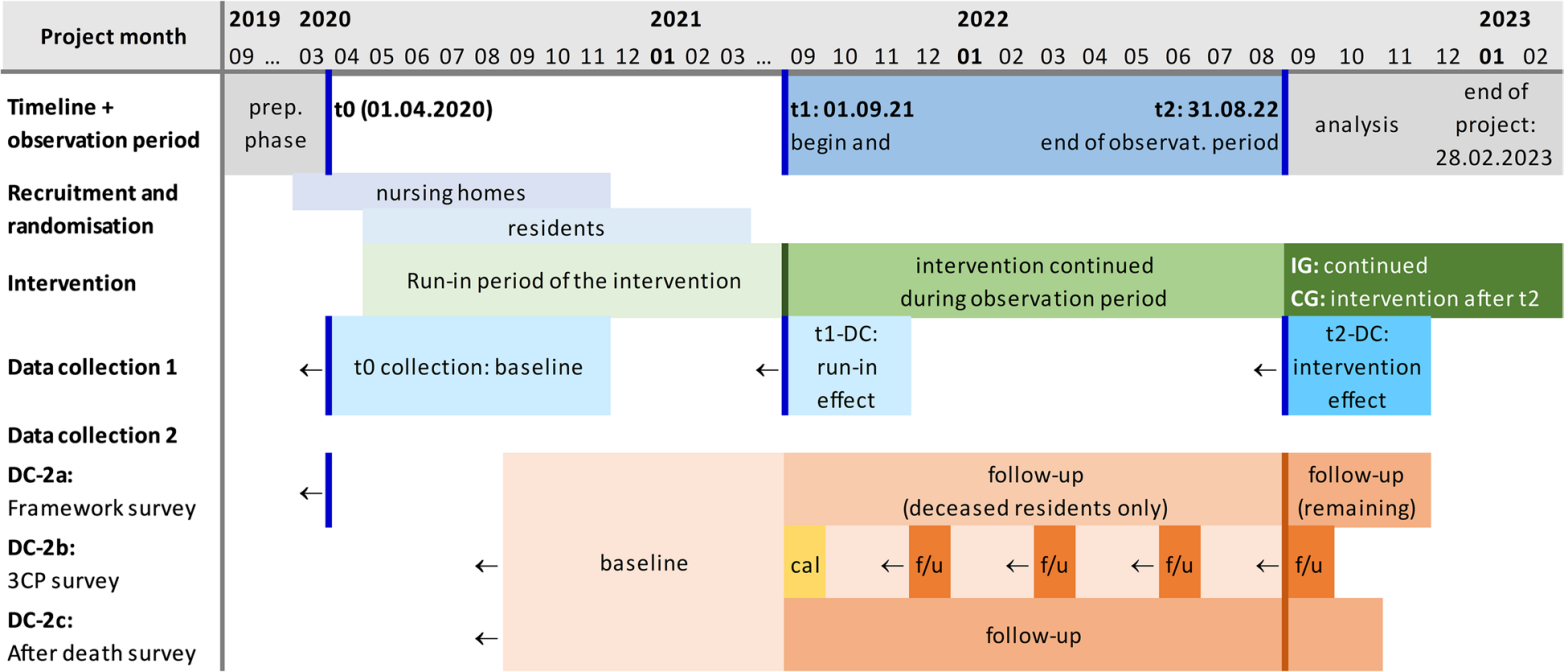
Schedule of enrolment, interventions and assessments after pandemic-related extension of project duration (update 05/2021). *Caption:* *IG* intervention group, *CG* control group, *f/u* follow-up. **“Cal”** denotes a trial data collection circle for calibration purposes, referring to 3 months before t1. *T0* is reference point for the retrospective parts of the baseline data collection 1 (DC-1) and data collection 2a (DC-2a); *t1* highlights the end of the run-in period of the intervention and the beginning of the observation period; *t2* marks the end of the observation period. *DC-1* consists of two parts: (a) a retrospective NH records survey referring to the 12 months prior to t0, t1, and t2, respectively, and (b) a cross-sectional NH records survey at the day of data collection. *DC-2* consists of three parts: *DC-2a (framework survey)* is a cross-sectional NH records-based survey at the day of collection combined with a retrospective NH records survey up to 12 months prior to t0 and t2, respectively; *DC-2b (care consistency with care preferences (3CP))* is a retrospective, NH records- and interview-based evaluation of critical treatment decisions 3 months prior to data collection; *DC-2c (after death survey*) is an interview survey of the bereaved person and responsible nurse

Originally﻿, this study was designed to last 3 years from its beginning in September 2019. After a 6-month preparatory phase, randomization and intervention were to start at month 7 (i.e., March 2020), followed by a 9-month run-in period of the intervention and a 12-months observation period. During 03/2020 to 03/2021, however, the SARS-CoV-2 pandemic restricted the NHs’ capacities for participation to times almost zero. Therefore, NH recruitment and randomization were extended until 11/2020 in order to compensate for SARS-CoV-2 related withdrawals. In 05/2021, when NHs had become accessible again 2 to 3 months after complete vaccination of NH residents, it was decided to modify the original timeline: The start of the observation period was postponed by 9 months in order to allow for a robust run-in period of the intervention. The observation period remains 12 months (now 09/2021 to 08/2022), preceded (in the intervention group) by a variable run-in period of the intervention of 9 to 17 months, depending on the time of randomization. In Fig. 3, we depict the timeline after the pandemic-related changes in the run-in period.

### Sample size {14}

We presume an annual hospitalization rate of 48% per year [40]. With our intervention, we expect to reduce this rate by absolute 8.6 to 39.4%. The calculation is based on a test for two proportions in a cluster-randomized design to detect a difference in the hospitalization rate during the 12-months observation period between intervention and control group. With an average size of 80 resident beds per NH and a postulated intra-cluster correlation of 0.03, we require 44 NH (44 × 80 = 3520 resident beds in total) to detect a statistically significant absolute reduction in the hospitalization rate from 48 to 39.4% with a power of 80% and type-1 error of 5% (two-sided). With a conservatively presumed death and moving rate of 25% per year, data of an average of 100 residents per home (44 × 100 = 4400 residents in total) will be used. To compensate for a presumed dropout of four NHs, 48 NHs (≈ 3840 resident beds) have to be recruited. Calculations were performed with the software PASS 16.0.3.

### Recruitment {15}

#### Recruitment of nursing homes

With the support of established regional networks, NH recruitment will be performed in three steps:

First, the NHs will be invited to participate in the study by phone, e-mail, fax, or in a personal conversation (e.g., in the context of meetings). The written invitation will be accompanied by information about the study. Second, a personal or telephone conversation will be held on the basis of the sent information. In NHs interested in participation, details of the cooperation agreement will be discussed in a personal meeting. And third, all participating NHs receive an expense allowance for data collection: (three times × 8 h + eight times × 2 h) × € 30 per hour. For further details of the recruiting process, see checklist in additional file 3.

For the recruitment of residents, representatives, relatives, nursing staff, ACP facilitator and coordinator, and regional player, see section “Who will take informed consent? {26a}”

## Assignment of interventions: allocation

### Sequence generation {16a}

A stratified blockwise 1:1 cluster randomization with variable block lengths will be performed at the level of NHs (= cluster). Based on a list of computer-generated random numbers, NHs will be randomly assigned to the intervention or control group, stratified by study site and region.

### Concealment mechanism {16b}

The Coordination Center for Clinical Trials at the Heinrich Heine University of Düsseldorf (KKSD), as a neutral partner, will receive the randomization lists directly from a study independent researcher of the Institute of Medical Biometry and Epidemiology of the University Medical Center Hamburg-Eppendorf. This person is neither involved in planning nor the analysis of the study. Nobody else will be informed about the details of the list. The KKSD will keep the list concealed until implementation of the random allocation and communicate the results of the randomization via email to an authorized member of the respective study center’s intervention team.

### Implementation {16c}

The stratified, sequentially numbered randomization lists will be generated by an independent researcher affiliated to the Institute of Medical Biometry and Epidemiology of the University Medical Center Hamburg-Eppendorf using R version 3.6.3. The allocation will be implemented by the neutral study partner KKSD after (a) recruitment of the NHs (clusters) by the study centers’ intervention teams, (b) baseline data collection 1, and (c) first wave of resident recruitment for data collection 2 have been completed by the study centers’ data collection teams. The study centers’ intervention teams will notify NHs of the outcome of the randomization.

## Assignment of interventions: blinding

### Who will be blinded {17a}

Biometricians will be consequently blinded until final data analysis. The statistical analysis plan will be finalized before unblinding. Data collection teams will be blinded to the allocation of NHs to intervention versus control group. The aggregated anonymous data from data collection 1 will be directly, i.e., without involvement of the data collection team uploaded by NH staff to our external data management partner KKSD (see below: data collection). With regard to data collection 2, to safeguard blinding in the crucial process of assessing care consistency with care preferences (3CP) by appreciating and synthesizing the available data sources, the case charts created for every single treatment decision (including relevant chart extracts and the pertinent interviews as audio files) will be assessed for 3CP not by the respective data collection team but by a peer team of one of the other three BEVOR study centers who have no contact with or knowledge of the NHs of the other study centers. Participating NHs and residents cannot be blinded due to the nature of the complex ACP intervention.

### Procedure for unblinding if needed {17b}

As participating NHs and residents cannot be blinded, there are no provisions for unblinding in the study.

## Data collection and management

### Plans for assessment and collection of outcomes {18a}

A contact person in each of the participating NH will receive a 1-h training and a manual for the two separate data collections (for details see Table 1, for the timeline of the data collection see Fig. 2):

#### Data collection 1: anonymous complete survey

The data will be collected by the NH contact person from the NHs' routine data and residents’ files, supported by the study team on site, which has received at least a 2-h training for data collection. All data will be aggregated on NH level, exported, and uploaded to the KKSD server by the NH contact person. Data will be checked and fed into the electronic case report form (eCRF) by the data manager of the KKSD.

#### Data collection 2: survey of subset of residents who gave informed consent

All residents living in the participating NHs at t0 will be asked for consent to a collection of detailed data from their chart records. The data collection teams will receive regular training to ensure data quality. In particular, data collection teams of the four study centers will together be extensively trained for employing the 3CP tool: 3-h theory and introduction to the excel-tool, 3-h simulated-patient training for the interviews, 4-h on-site training for identifying potentially life-threatening events in the charts, 6-h self-study to practice integrated judgment, and fortnightly 2-h group meetings until sufficient calibration is achieved in every step of applying this complex tool. Also, leading the After Death Bereaved Family Member Interview will be trained in a theory workshop and with simulated patients in small groups, which sum up to 5 h.

The data collectors will extract data from NH files (paper or electronic based), and interview residents, relatives, surrogates, and NH staff on-site or via telephone. For surveying quality of life, they lead or organize questionnaire surveys with residents, caregivers, and relatives, taking into account the local conditions. General practitioners, EMS, and hospital controlling will be contacted via email, fax, and/or phone and asked to transfer the required data. Support in data collection is offered on-site. Collected data will be entered into the electronic case report forms (eCRF) (see below: “Data management {19}”).

#### Data collection for the health economic evaluation

In addition to the consumption of healthcare services described by data collection 2, time expenses to deliver the ACP intervention will be documented continuously by the intervention study team and by external active supporters of the intervention like regional ACP coordinators.

#### Data collection for the process evaluation

Data collection comprises guideline-supported interviews one-to-one or by telephone with nursing home management staff and nursing staff from each NH of the intervention group (*n*=22), ACP facilitators, emergency medical service staff, general practitioners, and other stakeholders from the local health system. Coordinators from all four study sites will be invited to a group discussion after the intervention period. Questionnaires will be distributed anonymously to gather information from staff, residents, and family from the NHs. Written progress documentation will be used for data collection from facilitators and coordinators to inform intervention delivery.

### Plans to promote participants retention and complete the follow-up {18b}

NHs receive financial compensation for the data collection they conduct (three times 8 h + eight times 2 h at € 30 per hour). Furthermore, NHs of the control group will receive the intervention after the observation period.

### Data management {19}

Data management will be performed according to the Standard Operating Procedures of the Coordination Centre for Clinical Trials Düsseldorf (KKSD). Procedures include development of electronic case report forms (eCRFs), implementation of validity and consistency checks, and database validation and documentation. The KKSD provides training for study site staff and supports data quality control and query management. Procedures are detailed in the Data Management Plan. The eCRF will be implemented in a Clinical Data Management System with Electronical Data Capture functionality available at the KKSD, TrialMaster^TM^ from Anju Software. Access to the computer system is restricted, adequate data backup procedures will prevent data loss. Only authorized persons may enter or access data in the study database based on predefined roles. The data will be stored for 10 years after the end of the project and will be deleted afterwards.

#### Data management for process evaluation

Interviews will be audiotaped and transcribed verbatim. Audio data will be deleted as soon as possible, transcripts will be stored securely. Paper-based anonymous questionnaires as well as informed consent forms or logbooks will be stored in a lockable filing cabinet in a room not accessible to the public. All requirements of the current data protection concept will be followed.

### Confidentiality {27}

Collected data will be protected in accordance with European Union data protection standards and national data protection regulations. Data of collection 1 will be completely anonymous. Data of collection 2 will be recorded, stored, and evaluated in audio recordings, digital photographs, on paper, and on electronic data carriers in a pseudonymized form. The pseudonymization is performed immediately on site. The pseudonymization codes are stored separately and independently of the project data on local multiple-protected university servers at the four study sites, only accessible to the local data collectors. For safety and monitoring purposes, a print copy of the pseudonymization code is safely stored in the investigation site file in the institute. Most data are stored at the KKSD and a smaller part at the four study centers where they are protected against loss and access by third parties in accordance with current IT security standards.

The Institute of Medical Biometry and Epidemiology is responsible for data analysis. Only involved employees have access to study data. Data and other documents produced in the course of the analysis are stored on a password-protected server and are archived for 10 years after the end of the study. Personal data will be anonymized as soon as this is possible according to the research purpose.

### Plans for collection, laboratory evaluation, and storage of biological specimens for genetic or molecular analysis in this trial/future use {33}

There will not be any biological specimens collected in the study.

## Statistical methods

### Statistical methods for primary and secondary outcomes {20a}

For both, *primary and secondary outcomes* measured at t0 (baseline before intervention) and t1 (begin of observation period), descriptive statistics will be presented separately for the two treatment groups, i.e., intervention and control, on NH level as well as on resident level. Categorical variables will be summarized by absolute and relative frequencies. Continuous variables are summarized by mean and standard deviation or by median, quartiles, and/or interquartile range, as appropriate. The number of available observations and the number of missing observations will be reported separately for the intervention groups. Tests of statistical significance will not be undertaken for baseline characteristics; rather, the importance of any imbalance will be noted.

In the main analyzes, the primary and secondary outcomes will be evaluated according to the intention-to-treat principle and will be based on all available data of all included nursing homes. The primary outcome is defined as hospitalization rate per 100 residents per year during the observation period (t1–t2), measured at the NH level, and will be compared between intervention and control group. For the rate comparison, a Poisson regression model will be calculated taking into account the group (intervention vs. control), realized follow-up time as offset, study center (and region if more than one), and the respective baseline rate as independent variables. The resulting statistical test for group comparison of intervention and control group will be performed two-sided at the 5% significance level. For sensitivity analysis, an evaluation with the negative binomial distribution to detect a possible but not expected over-dispersion will be conducted.

The evaluation of secondary outcomes will be performed in an exploratory manner without adjustment for multiplicity. All analyses relating to the residents of care facilities will be performed within a framework of multi-level models, by including a random intercept for the care facility. For endpoints corresponding to a rate comparison, mixed Poisson regression models will be calculated with number of events up to t2 as the dependent variable, group (intervention vs. control), and study site as fixed effects, realized follow-up time as offset, the particular baseline measurement as a covariate, and care facilities as random effect. Similarly, changes in other endpoints will be analyzed in accordance to their statistical distribution by using appropriate regression models. For binary outcomes, a mixed logistic regression model will be calculated, taking into account the respective binary outcome as dependent variable, group (intervention vs. control), and study site as fixed effect and care facilities as random effects. For continuous outcomes, a linear mixed model will be calculated including change from baseline (t2–t0) as dependent variable, group (intervention vs. control), and study site as fixed effects, the respective baseline value as covariate, and care facilities as random effect. Additional per protocol analyses will take into account whether a minimum share of residents had ACP conversations in the respective nursing home.

Secondary outcomes measured at care facility level will be analyzed according to their statistical distribution by using appropriate regression models—Poisson regression models for count data in terms of rate comparisons, logistic regression models for binary outcomes, and linear regression models for continuous outcomes. A detailed statistical analysis plan will be prepared and finalized before unblinding and start of the analysis. Standard statistical software such as STATA (Version 16.0 or newer), R (Version 4.0.5 or newer), SAS (Version 9.4 or newer), or SPSS (Version 25 or newer) will be used for the statistical analyses.

#### Health economic evaluation

The objective of the health economic evaluation is twofold: first, to get an overview of all cost items and outcomes to understand the potential impact of the intervention on both in a cost-consequence analysis, and secondly, to assess the cost-effectiveness of the intervention in a cost-effectiveness analysis. In both the cost-consequence analysis and the cost-effectiveness analysis, the cost and outcome of the intervention group (treatment according to the ACP approach) is compared to the cost and outcome of the control group (usual care). All costs resulting from the consumption of healthcare services as well as costs associated with the intervention are considered from the perspective of the German social insurance (statutory health insurance and long-term care insurance).

In the cost-consequence analysis, all cost items and outcomes are listed separately by group assignment. In contrast to the cost-consequence analysis, cost items are aggregated and a cost-outcome ratio is calculated in the cost-effectiveness analysis, i.e., the incremental cost-effectiveness ratio in terms of (I) additional costs per additional hospital admission averted and (II) additional costs per additional case of care consistency with care preferences.

The incremental cost-effectiveness ratio is determined on cluster level as the ratio of the difference in mean costs and difference in mean outcomes between intervention and control group. A general linear model is used to adjust for baseline covariates.

The main analysis will be conducted according to the intention-to-treat principle. Missing cost and outcome values will be replaced by using multiple imputation by chained equations [49, 50]. A complete case analyzes including study participants with available data only is performed in sensitivity analysis to assess the potential influence of missing values in the dataset. Due to the short study period, no discounting of the outcomes and costs is required. A 95% confidence interval for the incremental cost-effectiveness ratio will be obtained using the non-parametric bootstrap method [51]. Uncertainty will be assessed by performing univariate sensitivity analyses and calculating cost-effectiveness acceptance curves [52, 53].

#### Process evaluation

We will analyze qualitative data from the interviews and group discussion using MAXQDA [54]. Since the interviews are guided by the core constructs of the Normalization Process theory, we will code the data directly via the headings of the various NPT constructs and components [48]. Quantitative data derived from questionnaires will be analyzed descriptively using IBM SPSS 25. Databases will be created with EpiData 4.6.0.2. Free text answers will be evaluated by qualitative content analysis [55]. Results of qualitative and quantitative data will be related to answer the proposed research questions.

### Interim analyses {21b}

No interim analyses are planned.

### Methods for additional analyses (e.g., subgroup analyses) {20b}

Additional analyzes will be detailed in the statistical analysis plan, which will be prepared and finalized before the start of the analysis.

### Methods in analysis to handle protocol non-adherence and any statistical methods to handle missing data {20c}

The number of available observations and the number of missing observations of each variable as well as major protocol deviations will be presented for the intervention and control group separately, as well as for the total sample. All data will be analyzed in an intention-to-treat approach. In case of substantial amount of protocol deviations, a per-protocol (PP) analysis using only data of NHs without protocol deviations will be performed as sensitivity analysis.

The main analysis strategy is using all available data of all randomized NHs (full analysis set). For the primary endpoint (and all secondary endpoints related to data collection 1), no missing data is expected due to the nature of aggregated data collection. For the unlikely situation of missing values, a multiple imputation using chained equations will be applied for a sensitivity analysis. The analysis of secondary endpoints on resident level will be specified in the statistical analysis plan.

### Plans to give access to the full protocol, participant-level data, and statistical code {31c}

There are no plans for public access.

## Oversight and monitoring

### Composition of the coordinating center and trial steering committee {5d}

The trial steering committee is composed of the institutional local investigators of the consortium and the project coordinator. The Coordinating Center for Clinical Studies of the Heinrich Heine University of Düsseldorf (KKSD) is an external partner responsible for data management and monitoring.

### Composition of the data monitoring committee, its role and reporting structure {21a}

Data monitoring will be performed by the KKSD, a neutral and independent partner (see above, item 16). A data monitoring committee will not be implemented due to the low risks of the intervention. Furthermore, it was not required by the ethical committees of the four study centers.

### Adverse event reporting and harms {22}

ACP facilitators are trained to detect any emotional disturbances during or following the ACP process, and to deal with them adequately, where necessary by involving other available resources such as NH staff, treating physician, priest, palliative care specialists, or ACP experts of the study team. NH staff will be encouraged to support this detection and reporting process. Adverse events will be reported.

In addition, residents may (not) receive life-sustaining treatment in accordance with their written advance care plan in place that does not, however, reflect their true care preferences. This risk is immanent to the setting, though, and altogether reduced, not increased, by implementing a complex regional ACP program.

### Frequency and plans for auditing trial conduct {23}

The KKSD provides external, independent monitoring of the trial in order to ensure that it will be conducted according to protocol. The monitor will verify the accuracy of the informed consent forms and, in direct cooperation with the NHs, existence of the named residents.

The data collection teams at the four study sites will perform a source data quality control for data collection 2. In every NH, 10% of the data (or at least data of three residents) will be double-checked. Residents’ files will be selected randomly using R [56]. Transfer of collected data into the eCRF will be completely double-checked. Also, the monitor will control data entry into the eCRF.

### Plans for communicating important protocol amendments to relevant parties (e.g., trial participants, ethical committees) {25}

Amendments will be consented within the steering group, submitted to the ethics committees of the four study sites. Participants will, where pertinent, receive an additional information via the recruiting team.

### Dissemination plans {31a}

Results will be communicated via scientific and other publications, lectures, and presentations on (inter-) national congresses. Information of the public through diverse channels is an inherent part of the study.

## Discussion

The BEVOR trial is one of the few studies conceived to provide robust data on patient-relevant clinical outcomes in order to evaluate the effects of a complex regional ACP intervention focusing on NHs. We do not know of any other study that has attempted to evaluate a comprehensive systems approach to ACP implementation in a cRCT, combining interventions on the individual (resident, treating physician), institutional (NHs, EMS, hospitals), and regional (ACP coordination and networking) level. Since clusters are the NHs, not regions, institutional (EMS, hospitals) and regional interventions imply a risk of contaminating control NHs; however, we expect that without intervention on the individual and NH level, these interventions will not effectuate relevant changes on the patient (resident) level within a year or two.

At the same time, it is unquestionably ambitious to attempt a system redesign on such a large scale, intending no less than a fundamental cultural change with regard to respecting patients’ preferences in the context of medical treatment of life-threatening disease. Given the complexity of our intervention approach, process evaluation will play a central part in order to understand the contribution of single intervention elements to the observed effects, and of facilitators and barriers to successfully implementing ACP.

Finally, the intervention of this study was planned to start in March 2020, i.e., the very month in which the SARS-CoV-2 pandemic hit Germany and German NHs in particular. In this protocol, we have described the new timeline after adjustment, i.e., an extension of the run-in period of the intervention so that the latter could come into effect at all before the start of the 12-months observation period. Besides these adverse effects of the pandemic related to severely restricted access to the NHs, SARS-CoV-2 had also some impact on public understanding of prognosis in severely ill frail NH residents, and on awareness for the importance of advance care planning. Thus, it will be an unforeseen task in the discussion of the results of this study to appreciate how the SARS-CoV-2 pandemic influenced its results.

## Trial status

Protocol version number and date: 2022-05

Date recruitment began 01.04.2020

Date recruitment completed: 31.03.2021

Last patient out: 31.08.2022

Last visit: 30.11.2022

## Statement on timing of submission

Recruitment of NHs for the BEVOR study started in March 2020, i.e., the very month in which the Covid-19 pandemic hit Germany. Thus, while the necessity for ACP in NHs became more apparent for a larger public than ever before, the NHs’ capacity to cooperate with the study team as envisaged was severely reduced over the past 2 years, at long times to almost zero. Furthermore, tight infection control regulations restricted access to the NH over several months during the pandemic waves. At the same time, our study team’s resources were extremely challenged because almost every facet of the intervention, be it on resident, institutional or regional level, needed to be adjusted to the new situation and typically offered multiple times to eventually reach the respective target group. Finally, we succeeded to receive funding to extend the run-in phase of the intervention by another 9 months so that the observational period could be postponed by this time.

The enormous effort to overcome these challenges, to deliver the intervention despite all obstacles, and to save the study by realizing an extension (while funded extensions are against all customs) led to a delay in finalizing this study protocol. We submit now at a time when resident recruitment has been closed more than a year ago (last patient in: 31.03.21), and the end of the postponed 1-year observation period comes into sight (last patient out: 31.08.22).

## Supplementary Information

**Additional file 1:** BEVOR selected intervention details_V01f_2022-05-22.pdf. Further details of selected intervention components

**Additional file 2:** BEVOR TIDierR Checkliste_V01f_2022-05-22.pdf. Intervention description following the Template for Intervention Description and Replication

**Additional file 3:** BEVOR Checklist for nh recruitment_V02f_2022-05-15.pdf. Checklist used by study team during nursing home recruitment

**Additional file 4:** BEVOR Instruments for process evaluation_V03f_2022-05-15.pdf. Detailed description of the process evaluations’ instruments: data collection tool, purpose, target group, time point of data collection and content.

## Data Availability

The investigators will have common access to the final trial dataset as regulated in their consortial agreement.

## Abbreviations

ACP: Advance care planning
AD: Advance directives
ADBI: After Death Bereaved Family Member Interview
AD-P: Advance directives by proxy
BEVOR: German name of the trial: Patienten-relevante Auswirkungen von Behandlung im Voraus Planen: cluster-randomisierte Interventionsstudie in Pflegeeinrichtungen
CCCP = 3CP: Care consistency with care preferences
cRCT: Cluster-randomized controlled trial
DC: Data collection
eCRF: Electronic case report form
EMS: Emergency medical services
KKSD: Coordination Center for Clinical Trials Düsseldorf
NH: Nursing home
POLST-E: Physician’s Order for Life-sustaining Therapy in Case of Emergency
PP-analysis: Per-protocol analysis
RCT: Randomized controlled trial
SARS-CoV-2: Severe acute respiratory syndrome coronavirus type 2
