# Supplementary material for: Effectiveness of a complex regional advance care planning intervention to improve care consistency with care preferences: study protocol for a multi-center, cluster-randomized controlled trial focusing on nursing home residents (BEVOR trial)
Source: Trials. 2022 Sep 12;23:770. doi: 10.1186/s13063-022-06576-3 (PMC9465132; doi:10.1186/s13063-022-06576-3)

Additional file 1

Content

Additional file 1 ... 1
1 Qualification ACP-facilitator trainer ... 2
2 Qualification for non-physician ACP facilitators - Overview ... 3
2.1 ACP facilitator prequalification requirements ... 4
2.2 Recommended competencies for ACP facilitators ... 5
2.3 Assessment of facilitator performance ... 5
2.4 ACP facilitator qualification schedule ... 6
2.5 ACP documentation by Advance Care Planning Germany (selected sections) ... 9
2.6 ACP by proxy documentation of ACP documentation by Advance Care Planning Germany (selected sections) ... 10
3 ACP qualification of involved involved physicians ... 11
4 Steering groups in the nursing home (NH) ... 12
5 Qualification of nursing and social services staff ... 12
6 Regional ACP coordinator qualification ... 13
7 Regional ACP implementation: roles, player, institutions... **Fehler! Textmarke nicht definiert.**

This additional file 1 contains details on selected intervention components with focus on the ACP facilitation. The intervention is in accordance with the standards of the German ACP society, Advance Care Planning Germany.

In accordance to

ACP Advance Care Planning Deutschland

BEVOR trial is funded by

Gefördert durch: Gemeinsamer Bundesausschuss Innovationsausschuss

BEVOR

1 Qualification ACP-facilitator trainer

Module A

- **Formal requirement**: ACP facilitator qualification, letter of recommendation of ACP trainer
- **Objective:** 10 further ACP facilitation processes within the last 12 month, 3 supervisions by ACP Trainer of ACP facilitation by ACP trainer trainee, work-shadowing and co-trainer role in ACP facilitator qualification, eLearning (24 lessons)

Module B

- **Formal requirements**: Successful completion of module A
- **Objective:** prepare and hold keynote presentations of ACP facilitator qualification, simulation of training with simulated patients; skill-training: teaching and communication

Module C

- **Formal requirements**: Successful completion of module A and B
- **Objective:** Supervised teaching in ACP facilitator qualification, supervised one-on-one supervision of ACP facilitator trainee
- Final Feedback of ACP trainer

Certification only upon successful completion (standardized, cumulative assessment by the entire trainer team) of all three modules

Time and effort Module A: 72 les attendance + 30 les ACP-facilitation + 9 les supervised ACP-facilitation = 111 les

Time and effort Module B: 24 les eLearning + 40 les in 5 day-workshop

Time and effort Module C: 72 les attendance + 3 les preparation time + 4 les supervised ACP-facilitator-trainee-Supervision = 79 les

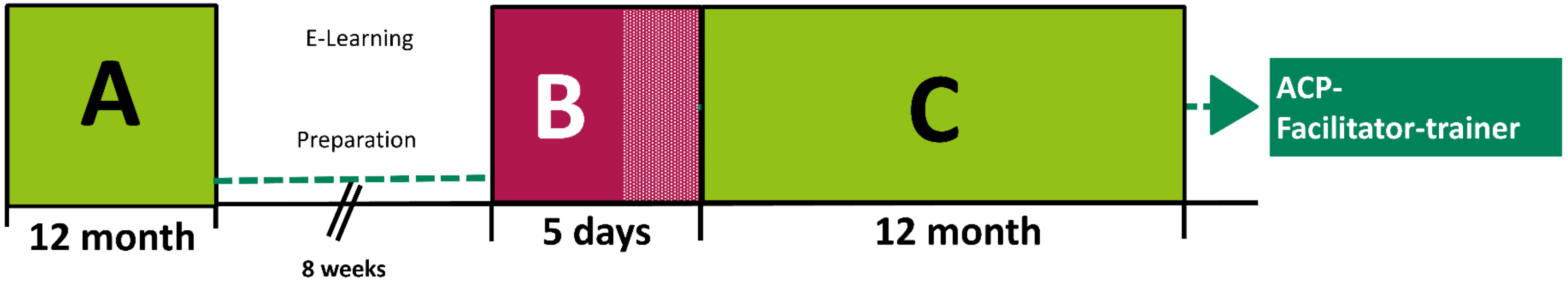

2 Qualification for non-physician ACP facilitators - Overview

ACP Advance Care Planning Deutschland

Module A (27 lessons (les) in 3 days): Basic course

- **Formal requirement**: non, open for interested people
- **Course objective:** In-depth understanding of ACP and, depending on the given prequalification and role, support of the institutional and/or regional implementation of ACP; successful completion is a prerequisite for further ACP facilitator qualification

Module B & C (45 les in 3+2 days): Advanced courses for the qualification of ACP facilitators (admission by trainer team)

- **Course goal:** ACP-facilitator according to agreement of 13.12.17 to § 132g SGB V
- **Formal requirements**: according to § 12, paragraph 4 of the agreement of 13.12.17 to § 132g SGB V and successful completion of module A
- Certification only upon successful completion (standardized, cumulative assessment by the entire trainer team)

Time and effort part 1: 72 (27+45) lessons attendance + 20 (12+8) lessons supervision of facilitation & coaching of documentation = 92 les

Time and effort Part 2: at least 4 lessons plenary meetings + at least 7*0,5 les coaching of documentation

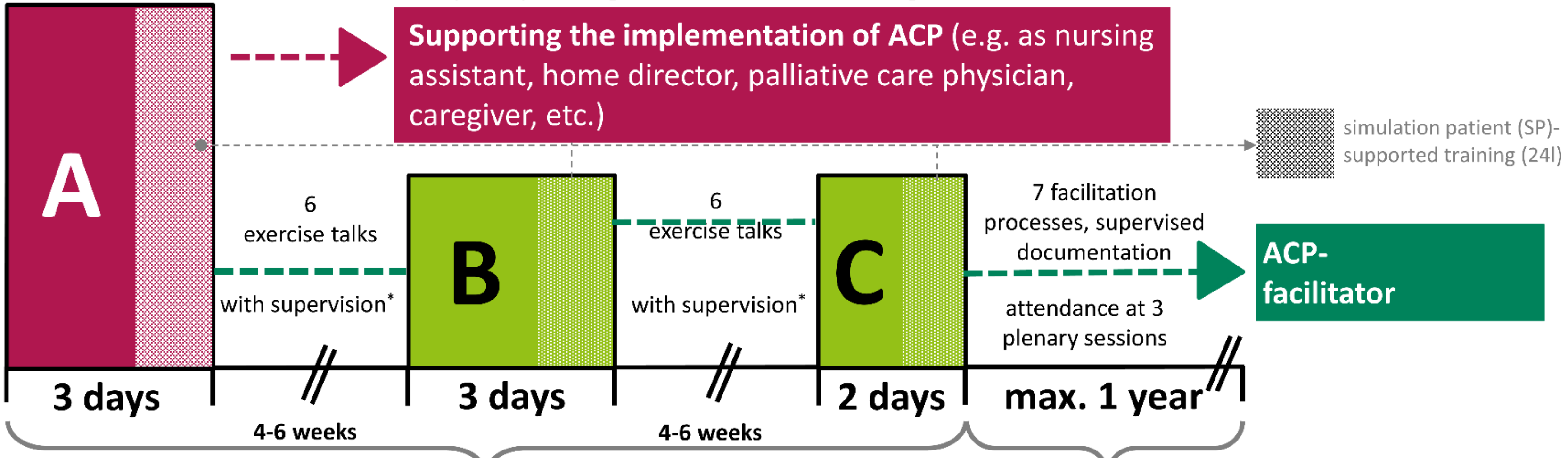

Part 1 according to § 12 of the agreement of 13.12.17 to § 132g SGB V

Part 2 according to agreement

* Four of the 12 sessions are supervised in the institution on site (12 les), the remaining eight are accompanied by telephone coaching based on the documentation (8 les).

2.1 ACP facilitator prequalification requirements

according to the agreement according § 132g Para. 3 SGB V on the contents and requirements of advance care planning for the last phase of life of 13.12.2017[1]

The facilitators have a basic qualification in the form of completed vocational training as

- nurse,
- geriatric nurse,
- pediatric nurse,
- state-approved curative education nurse
- state-recognized curative education teacher
- state-recognized nursery school teacher

or other comparable vocational training or

a relevant degree in the field of

- health and nursing sciences,
- humanities, social and educational sciences

(in particular as a pedagogue, curative pedagogue, social worker, social pedagogue, psychologist, psychotherapist, social worker, theologian)

and

three years of professional experience relevant to advance care planning within the last eight years, at least as a half-time position, in particular in a full inpatient care facility or an outpatient care service, an outpatient hospice service/outpatient children's hospice service (full-time coordinator), an inpatient hospice/inpatient children's hospice, an SAPV team, a palliative care unit or in a palliative service in the hospital or in institutions of the integration assistance for adults as well as children and adolescents.

[1] https://www.gkv-spitzenverband.de/media/dokumente/krankenversicherung_1/hospiz_palliativversorgung/versorgungsplanung/Vereinbarung_nach_132g_Abs_3_SGBV_ueber_Inhalte_und_Anforderungen_der_gesundheitlichen_Versorgungsplanung.pdf

2.2 Recommended competencies for ACP facilitators

according to the ACP task force of the German Association of Palliative Medicine in 2017

- ✓ An own view, acquired through corresponding professional experience, with regard to typical clinical pictures and crises in the institutions, in which the activity as a ACP-facilitator is to be taken up
- ✓ Experience in clinical documentation and competence in structured and precisely condensed recording of narratives
- ✓ Proof of successful participation in a course of at least 24 units "Fundamentals of client-centered interviewing (according to Rogers)" or a comparable qualification that uses participant-oriented methods to develop basic knowledge and skills, especially on the following topics (nota bene: is already part of many trainings and study programs):
    - empathic communication
    - non-directive interviewing, open versus closed questions
    - Addressing taboo topics
    - provoking narratives
    - taking and giving feedback
    - Picking up on hidden verbal and nonverbal conversational cues, noticing and picking up on channel discrepancies (verbal vs. nonverbal communication)
- ✓ Self-awareness, specifically: advanced self-reflection on end-of-life issues and potentially life-sustaining treatment, as well as one's own values in this regard
- ✓ Confident, appreciative demeanor towards colleagues and other staff members
- ✓ Respectful attitude towards different (especially: opposing) views and opinions
- ✓ German at native language level

2.3 Assessment of facilitator performance

A structured assessment is used during the ACP qualification and on-site ACP facilitation. The participant receives feedback after each module from all participating ACP trainers. There is one assessment for ACP facilitations with capable persons and one for ACP facilitation with proxies. Both are available on demand.

2.4 ACP facilitator qualification schedule

Programme of a standard ACP-facilitator Course

time	Module A: Basics, settings, emergency sheet				
	day 1 (9h) (8,6 TU)		day 2 (9h) (4,6TU)		day 3 (9h) (4,6 TU)
08.30	Introduction, course structure	08:30	SP-training: elements (I): "attitudes"	08:30	SP-training: elements (II): acute crises
09:15	Basics: Introduction to ACP, autonomy				
10:00	break	10:00	break	10:00	break
10:30	Conti. Basics: From conventional Advance Directive (AD) to ACP	10:30	SP-training: elements (I): "attitudes"	10:30	SP-training: elements (II): acute crises
12:00	**Elements der ACP-facilitation (I):** Conversation initiation, focusing	12:00	Reflection of SP-Training		Reflection of SP-Training
12:30	lunch	12:30	lunch	12:30	lunch (Trainers final conference)
13:30	**Conti. elements (I): "Attitudes"**	13:30	Elements ACP-facilitation (II): acute crises / POLST-E introduction	13:30	**"Recapitulation of the legal framework:** From Guardianship Law: Living Wills Act, ACP-instruments, tasks and selection of the representative. Consent and Legal capacity. HPG: §132 g SGB V.
14:15	Feedbackrules				
14:30	Conti. elements (I): Demo-Role-Play	14:30	Conti. elements (II): Demo-Role-Play		
15.00	break	15.00	break	15.00	**Last questions, homework, Closing of the course**
15:30	Conti. elements (I): Participant Role-Play in small groups	15:30	Conti. elements (II): Participant Role-Play in small groups	15:30	Individual Feedback for Participants
17.00	Closing day 1, Preparation of day 2	17.00	Closing day 2, Preparation of day 3		
17:30	End day 1	17:30	End day 2		

approx. 5 weeks until Module B, during that time:

--> At least 6 independently conducted "real" conversations in the facility (written documentation is a condition for admission to module B) Approval of the document by the trainer.

time	ModuleB: Acute inpatient treatment with incapcity of uncertrain duration and permanent decision-making incapacity				
	**Tag 1 (9h) (8,6 UE)**		**Tag 2 (9h) (8,3 UE)**		**Tag 3 (4h) (1,6 UE)**
08:30	Experiences from the past practical phase	08:30	Blitzlicht, Nachträge, Impulse	08:30	**SP-Training elements (III):**<br>**Acute inpatient treatment**
		08:45	**Forts. Elemente (III):**<br>TN-Rollenspiele in KG		
10:00	**break**	10:00	**break**	10:00	**break**
10.30	**Elements of ACP-facilitation (III):**<br>**Acute inpatient treatment with incapcity of uncertrain duration**<br>Introduction	10:30	**Conti. elements (III):**<br>documentation of facilitation process	10:30	**SP-Training elements (III):**<br>**Acute inpatient treatment**
		11:00	**Elements of ACP-facilitation (IV):**<br>**Dauerhafte Entscheidungsunfähigkeit**<br>Introduction		
		12:00	lunch	12.00	reflection des SP-training
12.30	lunch			12:30	lunch
		13:00	***Conti. elements* (IV):**<br>Introduction		
13:30	**Conti. elements (III):**<br>Einführung	13:45	***Forts.* Elemente (IV)**<br>Demo-Role-Play	13.30	**SP-Training: elements (IV):**<br>**Permanent decision-making incapacity**
		14.30	break		
15:00	break	15.00	***Conti. elements* (IV):**<br>Participant Role-Play in small groups	15.00	break (Trainers final conference)
15.30	**Conti. elements (III):**<br>Demo-Role-Play			15.30	**SP-Training: elements (IV):**<br>**Permanent decision-making incapacity**
16:15	**Conti. elements (III):**<br>Participant Role-Play in small groups	16:30	Closing day 2, Preparation of day 3		
17:00	Closing day 1, Preparation of day 2	**17:00**	**End day 2**	17.00	reflection des SP-training
**17:30**	**End day 1**			17.30	Closing day 3<br>Tasks till the next module
				**18.00**	**Individual feedback for participants**

approx. 5 weeks until Module C, during that time:

--> At least 6 independently conducted "real" conversations in the facility (written documentation is a condition for admission to module C) Approval of the document by the trainer.

time	Modules C: Representative's order and assembling the building blocks to form the "whole living will".				
	day 1 (9h) (8.6TU)		day 2 (9h) (4.6 TU)		day 3 (6h) (1.9 TU)
08.30	Experiences from the past practical phase<br><br>small groups and plenary	08:30	SP-Training elements (V):<br>Advance Care planning by proxy:	08:30	Vorbereitung SP-Training
				08:45	**SP-Training:**<br>Complete ACP-faciliation (proxy)
10.00	break	10.00	break	10:15	break
10.30	**Elements of ACP-facilitation (V):**<br>**Advance Care planning by proxy:**<br>Introduction incl. contra and pro. risks and dangers, legal framework	10:30	SP-Training elements (V):<br>Advance Care planning by proxy:		**SP-Training:**<br>Complete ACP-faciliation (AD)
		12.00	Reflection of SP-training	12:15	Reflection of SP-training
12.30	lunch	12:30	lunch	12:45	lunch (Trainers final conference)
13.30	**Conti. elements (V):**<br>Demo-Role-Play	13.30	**Consolidation and repetition of the complete ACP-Process: Conversation, questions,**<br>common obstacles, examples Participants<br><br>**(FAQ's)**	13:45	Closing GB-Workshop:<br>Vorbereitung des Teil 2 der Qualifizierung
14:15	Participant Role-Play in small groups			14:30	**Individual feedback for participants**
15.00	break	15:00	break		
15.30	**Conti. elements (V):**<br>Participant Role-Play in small groups	15:30	**Internal und external Networking:**<br>Einführung und KG		
		16:45	**Documentation acc. § 132g**		
17:00	Closing day 1, Preparation of day2	17:00	Closing day 2, Preparation of day3		
**17:30**	**End day 1**	**17:30**	**End day 2**		

2.5 ACP documentation by Advance Care Planning Germany (selected sections)

DiV-BVP ADVANCE DIRECTIVE © DiV-BVP e.V. 2020-05 | Germany | S. 3

General attitudes towards living, severe illness and death

Location Determination for finding therapy goals

How much do you enjoy living?
How strongly do you wish to live on (for a longer time)?

When you think of dying – what comes to your mind?
If I could tell you that you will peacefully fall asleep tonight and won't wake up again tomorrow morning - what would that mean to you now?

In a health crisis, would you allow your life to be saved by medical treatment? What burdens and risks would you be willing to accept? What worries or fears do you have about future medical treatment? What would you want not to happen at all?

Examples of situations in which you don't want to receive life-sustaining treatment.
Have you experienced yourself or other people to receive medical treatment against serious illness? If yes, what follows for your own future treatment, if anything?

Are there any religious, spiritual or personal beliefs or cultural backgrounds that are important to you in this context?

Date, Surname, First Name, Signature :

DiV-BVP Advance Directive © DiV-BVP e.V. 2020-05 | Germany | S. 7

Physician Order for Life-Sustaining Treatment in Case of Emergency (POLST-E)

Last Name ______ Adresse / ggf. Stempel der Einrichtung
First Name ______
Date of Birth __.__.____
Contact person in emergencies (ph.): :

In case of a life-threatening crisis or illness with loss of decision-making capacity, the following order applies: *Check only one answer box (A, B0, B1, B2, B3 or C) – otherwise invalid!*

GOAL OF TREATMENT = Prolonging life as far as medically acceptable

☐ **A** ●●●●● **Unlimited emergency and intensive care treatment with the goal of prolonging life, including cardio-pulmonary resuscitation (CPR)**

GOAL OF TREATMENT = Prolonging life, but with the following limitations

☐ **B0** ●●●●● **No cardio-pulmonary resuscitation (no CPR)** *Otherwise unlimited emergency and intensive care treatment*

☐ **B1** ●●●●● No cardio-pulmonary resuscitation (no CPR) **No invasive ventilation (i.e., no endotracheal intubation)** *Otherwise unlimited emergency and intensive care treatment*

☐ **B2** ●●●●● No cardio-pulmonary resuscitation (no CPR) No invasive ventilation (no endotracheal intubation) **No intensive care/no transfer to ICU** *Otherwise unlimited emergency treatment*

☐ **B3** ●●●●● No cardio-pulmonary resuscitation (no CPR) No invasive ventilation (no endotracheal intubation) No intensive care/no transfer to ICU **No transfer to hospital** *Otherwise unlimited emergency treatment*

GOAL OF TREATMENT = Palliation, not prolonging life

☐ **C** ●●●●● **Exclusively palliative care** *If possible remain at (residential) home.*

______, den ___ . ___ . 20___
„„I confirm capability, and understanding of the implications of this decision and medical acceptability."
☐ * Plan for medical crisis has been drawn.
x
Signature and stamp of certified **physician**

______, den ___ . ___ . 20___
„This POLST-E reflects my treatment preferences."
x
Signature of the **concerned individual**
„This POLST-E reflects the (presumed) preferences of the person."
x
Signature of **proxy**
„I have facilitated the advance care planning process."
x
Signature of the certified **ACP facilitator**

This ÄNo is also valid in the hospital as long as no deviating regulation is agreed upon there in accordance with the patient's will for a given reason (e.g. operation, intensive medical treatment or permanent inability to give consent).

DiV-BVP Advance Directive © DiV-BVP e.V. 2020-05 | Germany | S. 9

Hospital treatment in case of decisional incapacity of uncertain duration

In case of ongoing in-hospital treatment for life-threatening illness, and decisional incapacity of uncertain duration, the following shall apply:

GOAL OF TREATMENT= Prolonging life, as far as medically acceptable

☐ A ●●●●● Unlimited emergency and intensive care treatment

GOAL OF TREATMENT = dependent on determination of the patient's will by the representative

☐ B ●●●●● Limitation of life-prolonging treatment by my representative on the basis of the "attitudes" (p. 3) and, where appropriate, the further stipulations made here.

The limitations of the measures laid down in the Notice (p. 7) will continue to apply until my representative amends them, if necessary, to best represent my will in this situation.

Even if the occurrence of the following events or treatment results can be expected with a probability bordering on certainty (close to 100%), life-prolonging measures should not be continued / carried out:

Yes	Representative shall decide	No	**No, and I demand the omission / termination of life-prolonging measures even from a probability of occurrence of:**
			**Events / treatment results:**
☐	☐	☐	Permanent severe physical disability: bedriddenness, incontinence and constant need for nursing care (≥20% ≥40% ≥60% ≥80%)
☐	☐	☐	Persistent severe cognitive impairment: needs outside help to cope, forgets names, disoriented in time and place (≥20% ≥80%)
☐	☐	☐	Months of inpatient treatment (including rehabilitation if necessary) until my previous condition is regained (≥20% ≥80%)
☐	☐	☐	Death from (complications of) the current disease in the course of the disease, regardless of all further medical efforts (≥20% ≥80%)
☐	☐	☐	______ (≥20% ≥80%)

I accept the consequences of the probable death.

GOAL OF TREATMENT = Palliation, not prolonging life

☐ C ●●●●● **Exclusively palliative care.** An existing life-prolonging treatment should be discontinued regardless of the prognosis, accepting that death may occur!

Date, Surname, First Name, Signature :

DiV-BVP ADVANCE DIRECTIVE © DiV-BVP e.V. 2020-05 | Germany | S. 13

Treatment in case of permanent loss of decision making capacity

In case of an irreversible loss of the ability to decide, and an additional life-threatening crisis or condition, the following shall apply:

☐ *The following directive(s) shall apply also in case of a "persistent vegetative state" (and comparable situations), even if there remains a remote prognostic uncertainty.*

GOAL OF TREATMENT= Prolonging life, as far as medically acceptable

☐ A ●●●●● Unlimited emergency and intensive care treatment, incl. CPR

GOAL OF TREATMENT = dependent on determination of the patient's will by the representative

☐ B ●●●●● Limitation of life-prolonging treatment by my representative on the basis of the "attitudes" (p. 3) and, where appropriate, the further stipulations made here.

☐ Life-prolonging treatment only as long as I still enjoy living, according to my proxy's judgment

☐ If my state deteriorates to the condition described below, I reject from then on any life-sustaining treatment in case of an additional crisis (i.e., option C: palliative care only):

☐ **In any case shall apply:**

Nur 1 Kreuz!
- ☐ **No CPR** (POLST-E B0)
- ☐ No CPR **No invasive (=endotracheal) intubation** (POLST_E B1)
- ☐ No CPR No invasive (=endotracheal) intubation **No intensive care treatment** (POLST_E B2)

- ☐ No dialysis
- ☐ **No** continuous artificial feeding, e.g. via tube or vein
- ☐ No ______

It is the task of my representative to adapt the currently valid POLST-E (p. 7) and, if necessary, ongoing treatments with the aim of prolonging life over time in accordance with my above specifications!

GOAL OF TREATMENT = Palliation, not prolonging life

☐ C ●●●●● Exclusively palliative care.

This should also apply if I still enjoy life in the eyes of others!

Ongoing treatments aimed at prolonging life are to be discontinued at the risk of death, in particular the artificial supply of food and fluids.

Date, Surname, First Name, Signature :

2.6 ACP by proxy documentation of ACP documentation by Advance Care Planning Germany (selected sections)

DiV-BVP Advance Care Plan by Proxy © DiV-BVP e.V. | 2020-01 | p. 3

Attitudes towards life, serious illness and dying

Location determination for therapy goals

Current statements verbal and/or nonverbal
Advance Directive Statements in an AD
Treatment requests Earlier oral or written statements that contain specifications for specific situations.
Presumed will Based on concrete evidence (verbal/nonverbal statements or attitudes): "What would the person say if we could interview them today? (and how do we know)?"

What evidence is there on how much the represented person likes to live?
What meaning does it have for him/her to continue living (for a long time)?

What is known about what the person thinks or used to think about dying?
If she fell asleep peacefully tonight and didn't wake up tomorrow, what would that mean for her?

From the perspective of the represented person, may medical treatment help prolong his or her life in crisis? What burdens and risks would she be willing to accept for this?
Why is this the case for her/him? What is known about the represented person's concerns and fears regarding medical treatments?

IMPORTANT: If there are any indications of changed settings, this documentation must be updated!

Date, Surname, First Name, Signature (proxy) :

DiV-BVP Advance Care Plan by Proxy © DiV-BVP e.V. | 2020-01 | p. 5

Continuation of attitudes

Current statements verbal and/or nonverbal
Advance Directive Statements in an AD
Treatment requests Earlier oral or written statements that contain specifications for specific situations.
Presumed will Based on concrete evidence (verbal/nonverbal statements or attitudes): "What would the person say if we could interview them today? (and how do we know)?"

Are there known situations when the represented person no longer wanted life-prolonging treatment? Why is this the case with her/him? Are past experiences with her/him or other people with specific illnesses or treatments relevant in this context?

Are there any religious, spiritual, or personal beliefs or cultural backgrounds that are important to the person represented in this context?

(Further) comments on attitudes to life, serious illness and dying; here, if necessary, also open naming of ambivalences or contradictions, uncertainties and open questions:

Date, Surname, First Name, Signature (proxy) :

DiV-BVP Advance Care Plan by Proxy © DiV-BVP e.V. | 2020-01 | p.7

Physician Order for Life-Sustaining Treatment in Case of Emergency (POLST-E)

Surname, first name of the person unable to give consent: Address

Born on __.__.____

Representative, phone:

In case of a life-threatening crisis the following order applies for the above mentioned incapable person: *Check only one answer box (A, B0, B1, B2, B3 or C) – otherwise invalid!*

GOAL OF TREATMENT = Prolonging life as far as medically acceptable

- ○ **A** — **Unlimited emergency and intensive care treatment with the goal of prolonging life, including cardio-pulmonary resuscitation (CPR)**

GOAL OF TREATMENT = Prolonging life, but with the following limitations

- ○ **B0** — **No cardio-pulmonary resuscitation (no CPR)**; *Otherwise unlimited emergency and intensive care treatment*
- ○ **B1** — No cardio-pulmonary resuscitation (no CPR); **No invasive ventilation (i.e., no endotracheal intubation)**; *Otherwise unlimited emergency and intensive care treatment*
- ○ **B2** — No cardio-pulmonary resuscitation (no CPR); No invasive ventilation (no endotracheal intubation); **No intensive care/no transfer to ICU**; *Otherwise unlimited emergency treatment*
- ○ **B3** — No cardio-pulmonary resuscitation (no CPR); No invasive ventilation (no endotracheal intubation); No intensive care/no transfer to ICU; **No transfer to hospital**; *Otherwise unlimited emergency treatment*

GOAL OF TREATMENT = Palliation, not prolonging life

- ○ **C** — **Exclusively palliative care**; *If possible remain at (residential) home.*

__________, den ___ . ___ . 20___

„ This POLST-E takes into account the (presumed) will of the above-mentioned person to be treated and the medical justifiability."

○ * Plan for medical crisis has been drawn.

x
Signature and stamp of certified **physician**

__________, ___ . ___ . 20___

"This POLST-E takes into account the (presumed) will of the above-mentioned person for treatment."

x
Signature und NAME of **Representative**

„I have facilitated the advance care planning process."

x
Signature of the certified **ACP facilitator**

This ÄNo is also valid in the hospital as long as no deviating regulation is agreed upon there in accordance with the patient's will for a given reason (e.g. operation, intensive medical treatment or permanent inability to give consent).

DiV-BVP Advance Care Plan by Proxy © DiV-BVP e.V. | 2020-01 | p. 9

Therapy goal for future chronic deterioration of condition

☐ There are currently no indications of the (presumed) treatment intentions of the represented person in the event of a future chronic deterioration in condition. There is therefore no basis for corresponding advance planning. Therefore, in the future, decisions should be made according to the best knowledge and conscience of the represented person. (If necessary, no further entries on this sheet!)

GOAL OF TREATMENT = Prolonging life as far as medically acceptable

○ A — Lebensverlängernde Behandlung ohne Einschränkungen durchführen (übereinstimmend mit Option A in der ÄNo)

GOAL OF TREATMENT = dependent on determination of the patient's will by the representative

○ B — There are viable indications, documented in the attitudes toward life, serious illness and dying (pp. 3 and 5), of the (presumed) will to treat of the represented person, which justify the following concrete Advance determination justify:

In case of future occurrence of the following chronic, i.e. without prospect of improvement, deterioration of the condition (please tick applicable options):

○ Food / liquid can permanently no longer be swallowed sufficiently

○ Other future chronic condition exacerbation (please describe as specifically and vividly as possible):

further treatment should consistently follow an exclusively palliative therapeutic goal and, if necessary, exhaust palliative and hospice options for assistance in dying.

Previously initiated treatments with the aim of prolonging life should then be terminated - at the cost of subsequent death -, any measures to prolong life including the artificial administration of fluids and nutrition should be refrained from, and the previous POLST-E (p. 7) should be adapted accordingly (= option C).

GOAL OF TREATMENT = Palliation, not prolonging life

○ C — The represented person is already in a state in which life-prolonging measures should no longer be performed or should be discontinued (= POLST-E option C).

Date, Surname, First Name, Signature (proxy) :

3 ACP qualification of involved physicians

Module A (8 lessons (les) in 1 or 2 sessions): Basic course for certification

- **Formal requirement**: physician
- **Course objective:** In-depth understanding of ACP, POLSTE-E and general attitudes toward living, severe illness and death; the role and supervision of and correspondence with ACP facilitator; billing with health insurance according § 132g SGB V.
- Certification only upon successful completion

Module B, C and D (24 les): Advanced courses for Qualification for treating physician

- **Formal requirements**: Completion of module A
- **Course objective:** Basic communication skills for shared-decision-making, legal background, in-depth understanding of every part of ACP facilitation process incl. ACP by proxy and how to effectively proof the ACP-documentation.

Time and effort Module A: 3 les theoretical background + 1 les role play + 4 les simulation-patient role play

Time and effort Module B - D: 3*3 les theoretical background + 3*1 les role play + 3*4 les simulation-patient role play

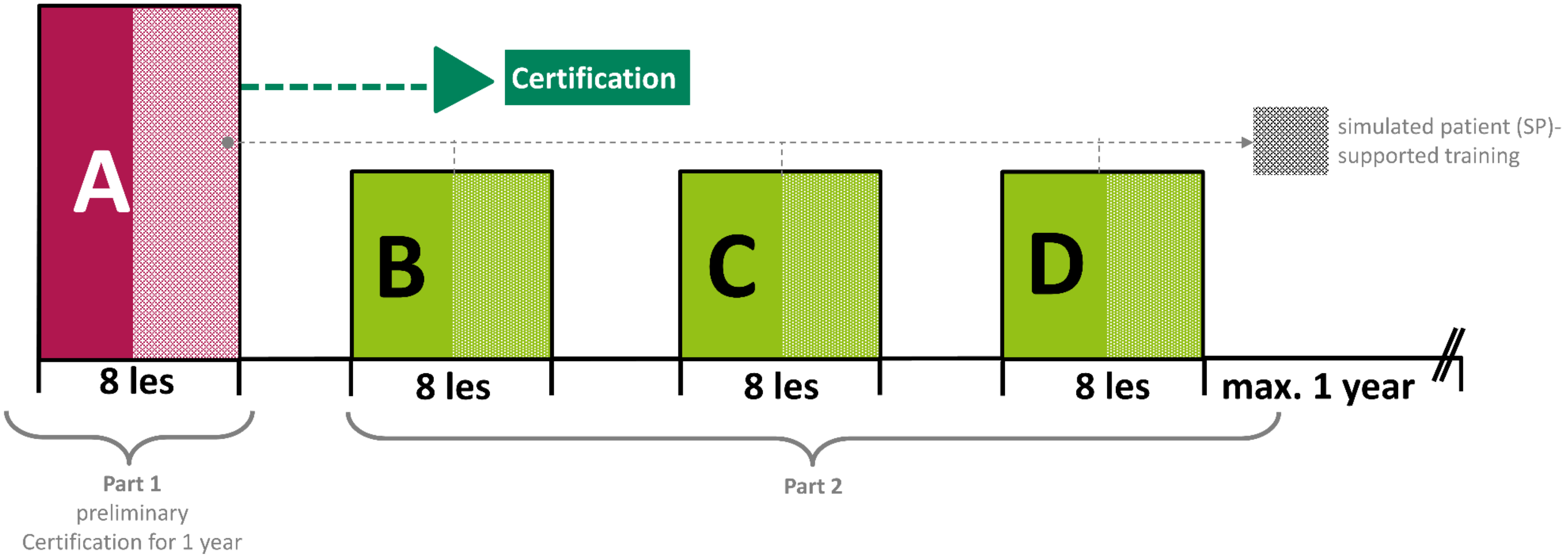

4 Steering groups in the nursing home (NH)

NH steering groups are responsible for the project management and system redesign, disseminating information about ACP and new procedural instructions, answering questions (e.g. staff, network partners, relatives) about ACP, being involved in the regional network with participation in network meetings, measuring and interpreting of quality indicators, writing reports for the head of the NH.

Members are the facility, nursing and residential area management, quality manager and ACP facilitator. The regional ACP coordinator or ACP trainer moderates the group.

5 Qualification of nursing and social services staff

Nr.	Duration	Content	Trainer
1	120 min	Q&A; ACP general information, AD and AD by proxy, facilitation process: general attitudes, POLST-E, role play (trainer plus attendant), permanent loss of capability	ACP trainer
2	45 min	AD in-depth	ACP trainer
3	45 min	AD by proxy in-depth	ACP trainer
4	45 min	AD and AD by proxy – roleplay or consolidation / Q&A	ACP trainer
5	45 min	Quality management: What changes for the staff?	ACP coordination
6	90 min	Telephone/contact with relatives in an emergency – role play	ACP trainer(s) + facilitator
7	45 min	Questions - Obstacles - Review	ACP trainer or facilitator
8	45 min	Case studies	ACP trainer
9	120 min	Taking up clues and deepening information from residents concerning ACP: role play	ACP trainer(s) + facilitator
10	30 min	Questions - obstacles – review - outlook	ACP trainer

Abbreviations: ACP advance care planning, AD advance directive, Q&A questions and answers, POLST-E physician order for life-sustaining treatment in case of emergency

6 Regional ACP coordinator qualification

- **Formal requirement**: ACP facilitator qualification, ACP trainer qualification is recommended
- **Objective:** Getting to know regional ACP system, stakeholder analysis, communicationplans with regional stakeholder; planning and managing a regional ACP project, systems re-design, skills: self-management, communication, managing

Time and effort: 7*3 les attendance + 7*3 les preparation and follow-Up = 42 les

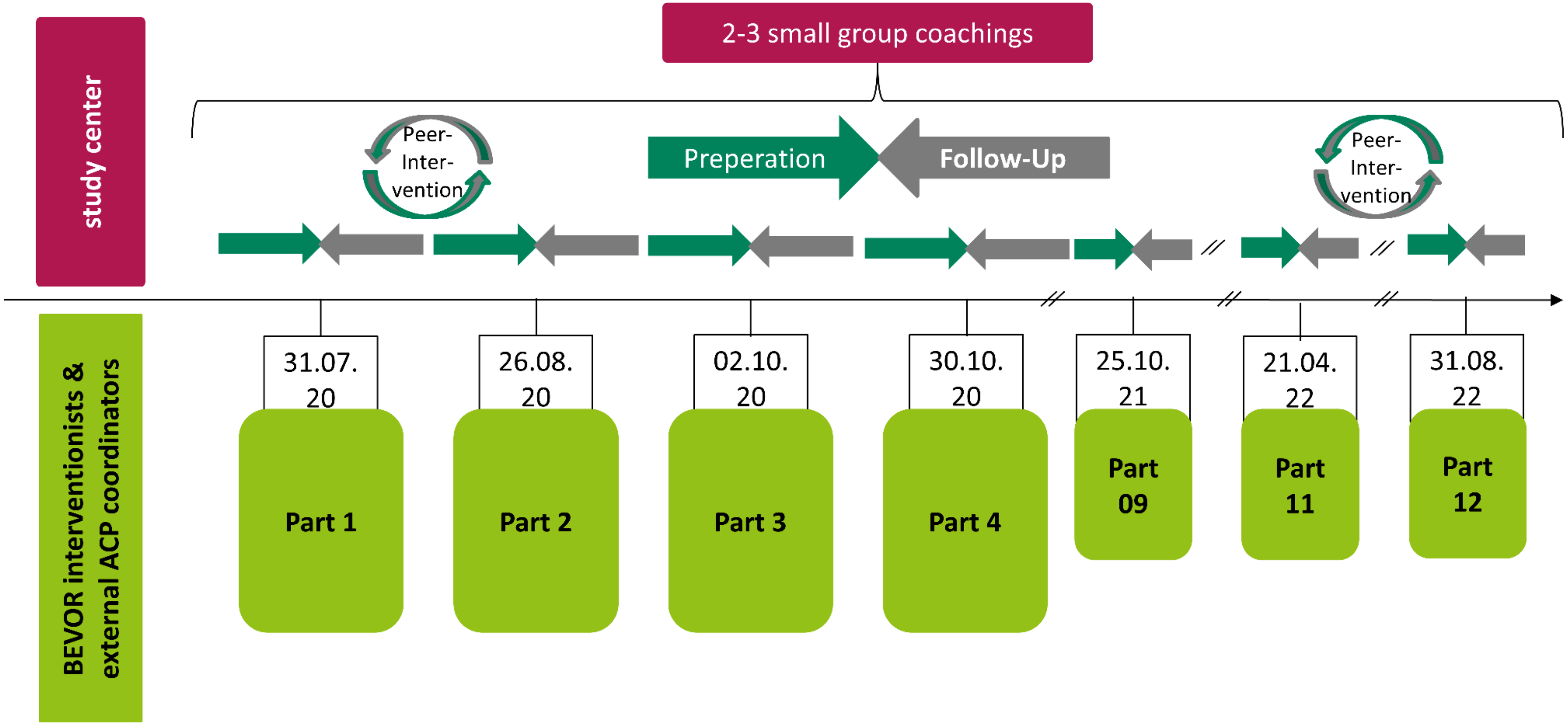

Supplement: Supplementary file 1 — Additional file 1: BEVOR selected intervention details_V01f_2022-05-22.pdf. Further details of selected intervention components [file 13063_2022_6576_MOESM1_ESM.pdf]
