# Supplementary material for: Effectiveness of a complex regional advance care planning intervention to improve care consistency with care preferences: study protocol for a multi-center, cluster-randomized controlled trial focusing on nursing home residents (BEVOR trial)
Source: Trials. 2022 Sep 12;23:770. doi: 10.1186/s13063-022-06576-3 (PMC9465132; doi:10.1186/s13063-022-06576-3)
Supplement: Supplementary file 2 — Additional file 2: BEVOR TIDierR Checkliste_V01f_2022-05-22.pdf. Intervention description following the Template for Intervention Description and Replication [file 13063_2022_6576_MOESM2_ESM.pdf]

TIDieR
Template for Intervention Description and Replication

The TIDieR (Template for Intervention Description and Replication) Checklist*:

Information to include when describing an intervention and the location of the information

Item Nr	Item		Where located ** Primary paper (page or appendix number)
1.	**BRIEF NAME** Provide the name or a phrase that describes the intervention.	Complex regional Advance Care Planning (ACP) intervention with a focus on nursing homes (NH), in accordance with the standards of the German ACP society, Advance Care Planning Germany.	---
2.	**WHY** Describe any rationale, theory, or goal of the elements essential to the intervention.	Overall goal of the intervention is to improve care consistency with care preferences in case of life-threatening illness accompanied by decisional incapacity. According to a theory of change (not reported yet), this requires interventions on the individual level (i.e., enabling individuals to develop and document preferences for critical treatment decisions in advance, supported by qualified ACP facilitators and treating physicians), on the institutional and regional level (i.e., implementing routines, educating all staff with view to attitudes, skills and knowledge relevant for ACP, warranting that the preferences of the person will be honored). For further rationales, see introduction of the study protocol.	study protocol p. 7
3.	**WHAT** Materials: Describe any physical or informational materials used in the intervention, including those provided to participants or used in intervention delivery or in training of intervention providers. Provide information on where the	*There is no open access to the materials. If you are interested in the qualification, please contact the authors.*<br>**Individual level:**<br>ACP trainers incl. their qualification[1]:<br>○ Preparation and follow-up: access to an online learning platform with a questionnaire on ACP; 2 incorrect ACP documentations and 2 videos of facilitated ACP conversations and an evaluation matrix to rate those conversations; 10 master PowerPoint slide sets covering all presentations for the ACP facilitator training incl. preparatory tasks to prepare for the face-to-face course and multiple-choice questionnaire, research articles, e.g. on resuscitation outcomes.	Study protocol Additional file 1

[1] Sections printed in orange were not immediate part of the trial, but describe necessary structural requirements of the intervention.

	materials can be accessed (e.g. online appendix, URL).	○ Face-to-face course: excel-tool for preparing small group allocation; process description of course units; forms for advance care plans (AD and AD by proxy); performance assessment tool for trainer and facilitator; trainer handbook, 12 simulated patient roles (simulation-patients (professional and lay actors) are standardized and need about 3 hours preparation time per role and 3 hours on site training by a simulation-patient-trainer (professional actor and director)<br>ACP facilitator qualification<br>○ Preparation: Information on the qualification and course schedule, schedule of (simulated patient) role plays, CME-article on ACP<br>○ Face-to-face course: 12 PowerPoint slide sets for presentations, 30 scripts on character and/or situation of simulation-patients for simulated patients role-plays, 6 scripts for participant role-plays; personalized forms for advance care plans (AD and AD by proxy); booklet “conversation aid”; information on resuscitation outcome data<br>○ Follow-up: Official documents on the reimbursement of the conversations (https://www.gkv-spitzenverband.de/media/dokumente/krankenversicherung_1/hospiz_palliativversorgung/versorgungsplanung/Vereinbarung_nach_132g_Abs_3_SGBV_ueber_Inhalte_und_Anforderungen_der_gesundheitlichen_Versorgungsplanung.pdf); template for ACP facilitation notes<br>ACP facilitation incl. advance care plans with supervision by treating physician<br>○ Personalized forms for advance care plans<br>○ Excel tool for traceability of the performed work<br>ACP qualification of treating physicians<br>○ Program flyer; cover letter; CME credit points; samples of ACP advance care plans (AD and AD by proxy); 16 scripts on character and/or situation of simulation-patients for simulated patients role-plays incl. incorrect documentation; 4 PowerPoint slide sets for presentations.<br>**Institutional level:**<br>NH steering group<br>○ Implementation manual with schedule, flowchart and templates; templates for application for financial coverage of facilitator employment by sickness funds; guidelines for kick-off meeting and further (up to 10) meetings<br>NH staff qualification<br>○ Description of qualification content and schedule; list of participants; PowerPoint slide set for presentations at kick-off meeting; 4 PowerPoint slide sets for training sessions, 4 guidelines for training sessions including 2 role-plays; handouts for communication with professionals and proxies in emergencies; guided self-reflection	

4.	**WHAT**<br>Procedures: Describe each of the procedures, activities, and/or processes used in the intervention, including any enabling or support activities.	in communication in emergencies; sample of advance care plans (AD and AD by proxy); ACP information folder for the NH wards<br>NH residents, relatives and surrogates<br>○ Print materials for distribution: brochure, flyer, poster; newsletter article; facilitator business cards of facilitator A5 with picture; 1 slide set to inform relatives and surrogates; guideline for face-to-face information of residents and relatives about ACP and the new NH's ACP policy<br>ACP qualification of emergency medical services<br>○ 2 PowerPoint slide sets; section in manual for further information; sample of POLST-E (physician's order for life-sustaining treatment in emergencies)<br>ACP qualification of hospital staff<br>○ 1 PowerPoint slide set; guideline for giving information on ACP to hospital staff<br>ACP qualification of other regional players<br>○ 1 PowerPoint slide sets adapted to the respective players´ interest and field of work<br><br>**Regional level:**<br>ACP-coordinator qualification<br>○ 15 timetable for participants and teacher; 13 PowerPoint slide sets; 5 home works; 5 communication-guidelines for respective regional player; briefing tool for emergency medical services; section in manual for further information, template for network contact list; template for documentation of network contacts<br><br>**Individual level:**<br>ACP facilitator trainer qualification:<br>○ ACP trainer selection and qualification of well before the start of the study<br>ACP trainer:<br>○ Search for and selection of sufficiently pre-qualified and appropriate individuals who are interested in ACP facilitation.<br>○ Supervision and support in the event of conflicts in the accompaniment of conversations<br>○ Planning and conducting voluntary follow-up facilitator meetings for in-depth training and peer-networking<br>○ Continued qualification in systems redesign, management, networking, implementation of ACP according<br>ACP facilitator qualification<br>○ Selection of sufficiently pre-qualified individuals	____________

		○ Training and supervision of ACP facilitator trainees<br>○ Planning and conducting plenary meetings over the time of qualification for in-depth training and peer-networking<br>ACP facilitation incl. ACP documentation<br>○ Getting acquainted with ACP relevant NH specifics, introduction to employees and treating physicians<br>○ Initiation and performance of ACP conversations with residents and surrogates/family<br>○ Exchange with treating physicians<br>○ Documentation in resident file and on specific forms for advance care plans (AD and AD-P incl. POLST-E); transfer of information and documents to NH staff; formal documentation for reimbursement<br>○ Participate in steering group meetings and staff trainings<br>○ Ongoing contact with ACP trainer/coordinator and other ACP facilitators to report and resolve challenges and facilitators<br>ACP qualification of treating physicians<br>○ Informing all treating physicians about ACP implementation in NHs<br>○ Invitation to participate in ACP training, planning of and conducting the online training including small group role play sessions with simulated patients<br>○ Continued availability of ACP experts for the event of critical challenges<br>ACP facilitator supervision by treating physician<br>○ Encouraging NH residents to make use of the offered ACP conversations<br>○ Discussing medical issues<br>○ Signing of the ACP documents, thereby attesting the capacity to consent and that the resident understands the implications of her documented preferences<br>**Institutional level:**<br>Steering group<br>○ Identify and positively decide on the necessary change management process<br>○ Ensure reimbursement and, if necessary, cooperation with regional ACP facilitator pool<br>○ Implement reliable processes of ACP conversations and of information transfer between facilitator and nursing team; develop and foster processes of continuous quality assurance; respond to ACP specific queries from employees; find solutions to ACP implementation barriers<br>○ Engage in the regional ACP network<br>Staff qualification<br>○ Arranging appointments with the implementing ACP experts and scheduling in the duty rosters	

		○ Preparation of the room and the documents for the qualification<br>○ Provision of an ACP information folder on the wards incl. telephone number of ACP-facilitator/trainer and coordinator<br>○ Execution of ACP handover by the ACP facilitator<br>○ Continued outreach by ACP experts to address barriers<br>**Regional level:**<br>ACP coordinator qualification<br>○ Select qualified individuals for planning and conducting the training<br>○ Scheduling, planning and performing the online training<br>○ Continued availability for ACP coordinators to address barriers and learn about facilitators<br>ACP coordination<br>○ Establish an ACP facilitator pool, a regional steering group and regional network with regular meetings<br>○ Public relation activities (e.g. flyers, posters, newspaper articles, website for the regional ACP project)<br>○ Support the NH in applying for reimbursement for ACP<br>○ Contact regional stakeholders to introduce ACP, conduct informational presentations<br>○ Ongoing contact with ACP trainers and ACP facilitators to resolve barriers and learn about facilitators<br>ACP qualification of emergency medical services, hospital staff and other regional players<br>○ Contact the medical director of emergency medical services, the medical director of the hospital, emergency department or any other person necessary to support ACP in the respective institution<br>○ Exchange about specific local conditions and agree on the qualification procedure and extent<br>○ Schedule and carry out the qualification<br>○ Continued availability of ACP coordinators to address barriers and learn about facilitators and invite the regional network	
5.	**WHO PROVIDED**<br>For each category of intervention provider (e.g. psychologist, nursing assistant), describe their expertise, background and any specific training given.	**Individual level:**<br>ACP trainer<br>○ ACP trainer with good ACP expertise, especially regarding the standards of Advance Care Planning Germany (ACP-D) and with ACP trainer-qualification (some of the trainer have completed their training with respecting choices (US) or respecting patient choices (Australia) because this did not exist in Germany before 2017); professional background in medicine, nursing, medical ethics<br>ACP facilitator qualification	study protocol p. 15 ff

		○ ACP trainer, certified with min. 250 h qualification according to the standards of ACP-D; professional backgrounds mostly in medicine, nursing, psychology, ethics, pastoral care, gerontology, or law.<br>ACP facilitation incl. advance care plans with supervision by treating physician<br>○ ACP facilitator with professional background mainly in nursing, social work or social pedagogics, but also paramedics, ethicists, psychologists, physicians, pastoral clergy and others (see additional file 1)<br>ACP qualification of treating physicians: ACP trainer who train the ACP trainer (see above)<br>**Institutional level:**<br>Steering group:<br>○ ACP trainer or coordinator/facilitator with further formal or informal qualification in institutional systems redesign<br>Staff qualification:<br>○ ACP trainer or facilitator/coordinator with further qualification in institutional systems redesign, and didactic, methodical transfer of ACP relevant contents through ACP-trainer and ongoing support<br>Residents, relatives, and surrogates:<br>○ Mainly ACP coordinator/facilitator/trainer, in exceptions trained facility staff<br>**Regional level:**<br>ACP coordinator qualification:<br>○ 3 ACP trainer with high expertise in collaboration with a professional organisation developer, her professional background is business psychology<br>ACP regional coordination:<br>○ ACP coordinator with skills in systems redesign, management and networking skills, knowledge of German ACP specific legislation, and ACP expertise (preferably as facilitator or trainer)<br>ACP qualification of emergency service: ACP trainer or facilitator, optionally with paramedical qualification<br>ACP qualification of hospital staff: ACP trainer<br>ACP qualification of other regional players: ACP coordinator/trainer/facilitator	
6.	**HOW**<br>Describe the modes of delivery (e.g. face-to-face or by some other mechanism, such as internet or telephone) of the intervention	**Individual-level:**<br>ACP trainer qualification: job-shadowing; blended learning, individually, small groups and plenary sessions, face-to-face<br>ACP trainer: The mode of the trainer activity depends on the particular activity to be performed. All modes are available and adapted to the task at hand, e.g. face-to-face, groups, telephone, e-mail, letter, videoconference-tools	study protocol p. 15 ff

	and whether it was provided individually or in a group.	ACP facilitator qualification: blended learning, individually, small group and plenary sessions, face-to-face (online or on site) and telephone<br>ACP facilitation incl. advance care plans with supervision by treating physician: face-to-face (rarely online or by telephone), individually with the planning person (NH resident or their surrogate) and their relatives<br>ACP qualification treating physician: small group and plenary sessions, face-to-face (online or on site)<br>**Institutional-level:**<br>Steering group: group sessions; face-to-face (online or on site)<br>Staff qualification: group sessions, individually, face-to-face (on site and online)<br>Residents, relatives and surrogates: individually, small-groups, plenary sessions; face-to-face (online or on site), internet, mail, newsletter, posters, flyer, brochure<br>**Regional-level:**<br>ACP coordinator qualification: blended learning, small groups and plenary sessions; face-to-face (online)<br>ACP regional coordination: face-to-face (online and on site), telephone, Mail, computer work<br>ACP qualification of emergency service: small groups and plenary sessions; face-to-face (online and on site)<br>ACP qualification of hospital staff: small groups and plenary sessions; face-to-face (online and on site)<br>ACP qualification of other regionals player: small groups and plenary sessions; face-to-face (online and on site), internet, flyer, newspaper	
**7.**	**WHERE**<br>Describe the type(s) of location(s) where the intervention occurred, including any necessary infrastructure or relevant features.	**Individual level:**<br>ACP trainer qualification:<br>○ for example, academy of palliative care; sufficient space for dividing into small groups<br>ACP facilitator qualification:<br>○ various settings (e.g., academies for palliative care, universities, other educational academies); sufficient space for small group training, standardized simulated patients<br>ACP facilitation incl. advance care plans with supervision by treating<br>○ In the participating NHs or external facilitator offices; ACP conversations are performed in a room with confidential, secure environment; laptop, mobile, internet access, data backup with good data security, access to files of the NH residents<br>ACP qualification treating physician:<br>○ Face-to-face (mainly online in various settings (see facilitator qualification); supported by the regional medical council, CME credit points; standardized simulated patients	study protocol p. 15 ff

		**Institutional level:**<br>Steering group: NH, alternatively online with video conferencing software and sufficient devices to support active participation<br>Staff qualification: NH, alternatively online with video conferencing software and sufficient devices to support active participation<br>Residents, relatives and surrogates: NH, video conferencing software<br>**Regional level:**<br>ACP coordinator qualification: video conferencing software<br>ACP regional coordination: Eligible offices / meeting points in the region, depending on group composition<br>ACP qualification of emergency service: video conferencing software; emergency medical services station; public spaces; hospitals<br>ACP qualification of hospital staff: online; hospital; public spaces<br>○ ACP qualification of other regionals player: video conferencing software; public spaces; room of regional player; university	
**8.**	**WHEN and HOW MUCH**<br><br>Describe the number of times the intervention was delivered and over what period of time including the number of sessions, their schedule, and their duration, intensity or dose.	**Individual level:**<br>ACP trainer incl. their qualification: took place in 2017, 2018 and 2019, over about a year each<br>ACP facilitator qualification:<br>○ 8 times: Oct. 2019- Mar. 2020 and Sep. 2021 – Dec. 2021, 104h (24h with simulation patients), ongoing monthly plenary meetings online till Aug 2022<br>ACP facilitation incl. ACP documentation incl. supervision of treating physician:<br>○ 60 % of residents of intervention NH, Jun 2020 – Aug 2022; ≥ 2 conversations/resident à 60-120 min, ; ≥ 1 conversation/documentation with GP à 20 min<br>ACP certification treating physician:<br>○ 8 times: Oct. 2020 – Oct. 2021 and Nov 2021 – Jul. 2022, 4*8h (16h with simulation patients); ongoing individual support by ACP-trainer/coordinator/facilitator<br>**Institutional level:**<br>Steering group:<br>○ ~14 times in intervention NH: Jun 2020 – Aug 2021, 10 sessions à 60-120 min; Aug 2021 – Aug 2022 quarterly meetings à 60-120 min	Study protocol Additional file 1

		Staff qualification:<br>○ ~14 times in intervention NH: Jun 2020 – Aug 2021, 10 sessions à 60-120 min (2 general information for complete team, 10 intensive trainings for nurses and social worker); Aug 2021 – Aug 2022 1 session for complete team half-yearly, quarterly meetings for nurses and social worker and in case of occurring barriers at any time à 60-120 min.<br>Residents, relatives and surrogates information:<br>○ ~8 times and ongoing in intervention NH: Jun 2020 – Aug 2021 4 informational events for each group à 1h; flyer/brochure for each resident, Jun 2020: newsletter per Mail, poster on ward; Aug 2021- Aug 2022 quarterly informational events for each group à 1h; flyer/brochure for each new resident, newsletter half-yearly<br>**Regional level:**<br>ACP coordinator qualification: once, Apr 2020 – Aug 21, 12*3h + preparation<br>ACP coordination: 50%-part-time ongoing Apr 2020 – Aug 2022<br>ACP qualification of emergency service:<br>○ 8 times: 2 per region, Nov 2020- Aug 2021, 120 min; afterwards yearly<br>ACP qualification of hospital staff:<br>○ each hospital per region ~48 times: 6 per region, Nov 2020- Aug 2021, 120 min; afterwards yearly.<br>ACP qualification of other regionals player: tailored to demand in region, Nov 2020- Aug 2021	
9.	**TAILORING**<br>If the intervention was planned to be personalised, titrated or adapted, then describe what, why, when, and how.	Due to the great differences in institutional and regional structures and cultures, the intervention on institutional and regional level was designed to be tailored to give ample flexibility (e.g. day and daytime, but as well the format: plenary session with only one emergency service station or in the context of a symposium). Only the sequence of content delivered and the total amount of time was fixed. Due to the taboos touched by the intervention there is always the possibility to change the content to the upcoming barriers or emotional disturbances, and to deliver the regular content later on. The ACP facilitation process is also designed in such a way, i.e. that barriers or disturbances on the side of the residents, surrogates or relatives always have priority over the standardized course of the ACP conversation.	Study protocol p. 15 ff
10.‡	**MODIFICATIONS**<br>If the intervention was modified during the course of the study, describe the	Because of the SARS-Cov-2 pandemic, the ACP intervention needed to be considerably adapted due to severe restrictions (e.g. access restrictions to facilities and regional actors, limitation of the number of participants per training), shortness of staff in the facilities and other addressed regional institutions/services and illnesses /	study protocol p. 24 ff, figure 3

	changes (what, why, when, and how).	quarantines of the ACP facilitators. Thus, the intervention changed regarding group-size and setting (on site vs online), especially the period of time of delivery had to be stretched, as well as the time between training-sessions. The run-in-phase of the intervention, planned to last from Apr 2020 to Nov 2020, was extended to last until August 2021 (due to strong regional differences concerning the legal regulations and affectedness by SARS-Cov-2; the 12 months observation period and consolidation phase of the intervention was postponed by 9 months to Sep 2021 – Aug 2022.	
	**HOW WELL**	**N/A**	
**11.**	Planned: If intervention adherence or fidelity was assessed, describe how and by whom, and if any strategies were used to maintain or improve fidelity, describe them.		---
**12.‡**	Actual: If intervention adherence or fidelity was assessed, describe the extent to which the intervention was delivered as planned.	**N/A**	---

** **Authors** - use N/A if an item is not applicable for the intervention being described. **Reviewers** – use '?' if information about the element is not reported/not sufficiently reported.

† If the information is not provided in the primary paper, give details of where this information is available. This may include locations such as a published protocol or other published papers (provide citation details) or a website (provide the URL).

‡ If completing the TIDieR checklist for a protocol, these items are not relevant to the protocol and cannot be described until the study is complete.

* We strongly recommend using this checklist in conjunction with the TIDieR guide (see *BMJ* 2014;348:g1687) which contains an explanation and elaboration for each item.

* The focus of TIDieR is on reporting details of the intervention elements (and where relevant, comparison elements) of a study. Other elements and methodological features of studies are covered by other reporting statements and checklists and have not been duplicated as part of the TIDieR checklist. When a **randomised trial** is being reported, the TIDieR checklist should be used in conjunction with the CONSORT statement (see www.consort-statement.org) as an extension of **Item 5 of the CONSORT 2010 Statement.** When a **clinical trial protocol** is being reported, the TIDieR checklist should be used in conjunction with the SPIRIT statement as an extension of **Item 11 of the SPIRIT 2013 Statement** (see www.spirit-statement.org). For alternate study designs, TIDieR can be used in conjunction with the appropriate checklist for that study design (see www.equator-network.org).
