# Supplementary material for: Effectiveness of a complex regional advance care planning intervention to improve care consistency with care preferences: study protocol for a multi-center, cluster-randomized controlled trial focusing on nursing home residents (BEVOR trial)
Source: Trials. 2022 Sep 12;23:770. doi: 10.1186/s13063-022-06576-3 (PMC9465132; doi:10.1186/s13063-022-06576-3)
Supplement: Supplementary file 3 — Additional file 3: BEVOR Checklist for nh recruitment_V02f_2022-05-15.pdf. Checklist used by study team during nursing home recruitment [file 13063_2022_6576_MOESM3_ESM.pdf]

Additional file 3

Checklist: Nursing home requirements for participation in the BEVOR study

The nursing home (NH) management confirms by a signature that the following requirements are met, otherwise participation in the BEVOR study is not possible.

**NH and existing offers**	**signature**
The facility is not specialized exclusively on residents with only one specific illness (e.g. persistent vegetative state; psychiatric illnesses; protected area for people suffering from dementia)	
There is no existing regular offer of ACP-conversations in the sense of § 132 SGB V to all residents, covering – among other things – the question of a hospital admission in emergency situations	
There is no other offer to all residents to document in advance preferences about hospitalization in an emergency (e.g. POLST-E).	
The institution does not participate in other studies that could interfere with the BEVOR study and influence its results.	
There is a basic palliative care competence and hospice culture established in the NH, i.e. all employees in the institution know what is meant by palliative care and the hospice concept and support this; OR: The NH management plans to establish such a basic competence during the course of the study.	
There is a networking with cooperation partners/external palliative care services providers (outpatient hospice or palliative care services, general practitioners with palliative care qualification); OR: The NH management plans to establish such a network during the course of the study.	
**Support of the BEVOR study and implementation of ACP**	**signature**
NH carrier, home management and nursing service management explicitly welcome and support participation in the BEVOR study and the implementation of ACP that will accompany it in the short or medium term.	
Time, personnel and, if necessary, financial resources for data collection and intervention are available or receive priority.	
**Active participation in the ACP intervention in terms of § 132g SGB V**	**signature**
An implementation team is formed which, as an internal steering group, supports the implementation of ACP in the institution in cooperation with the (externally or internally employed) ACP facilitator and the study center.	
The facility employees are granted the possibility to participate in the information, training and reflection sessions regarding the internal implementation of ACP described in § 4 of the agreement.	
The ACP facilitator receives the necessary resources from the institution to be able to conduct ACP conversations with the residents and/or their representatives and, if necessary, their relatives within the scope of the intended job.	
The NH supports the information flow between the ACP facilitator and the caring personnel	

Funded by: Gemeinsamer Bundesausschuss Innovationsausschuss
