# Supplementary material for: Effectiveness of a complex regional advance care planning intervention to improve care consistency with care preferences: study protocol for a multi-center, cluster-randomized controlled trial focusing on nursing home residents (BEVOR trial)
Source: Trials. 2022 Sep 12;23:770. doi: 10.1186/s13063-022-06576-3 (PMC9465132; doi:10.1186/s13063-022-06576-3)
Supplement: Supplementary file 4 — Additional file 4: BEVOR Instruments for process evaluation_V03f_2022-05-15.pdf. Detailed description of the process evaluations’ instruments: data collection tool, purpose, target group, time point of data collection and content. [file 13063_2022_6576_MOESM4_ESM.pdf]

Gefördert durch:
Gemeinsamer Bundesausschuss
Innovationsausschuss

Additional file 4:

Instruments for process evaluation

Implementation

Data collection via	No	Purpose	Target group	Data collection	Content	Analysis
Documentation	1	Progress documentation	Study staff	Recruitment phase	Recruitment of nursing homes	Descriptive
Documentation	2	Progress documentation	Study staff	Recruitment phase	Recruitment of participants	Descriptive
Documentation	15	Progress documentation	Study staff (observer)	During data collection	Number of contacts with GPs	Descriptive
Log book	22	Intervention delivery	ACP facilitators	Continuously during intervention phase	Documentation of contact and progress	Content analysis
Log book	20	Progress documentation	ACP facilitators	Continuously during intervention phase	Monthly documentation of ACP-related activities and satisfaction over 18 months	Content analysis
Log book	21	Progress documentation, intervention delivery	ACP coordinators	Continuously during intervention phase	Monthly documentation of ACP-related tasks and activities over 18 months	Content analysis
Questionnaire	6	Characteristics	ACP facilitators	Baseline	ACP education; mode of employment, ACP working experience, level of preparation for ACP tasks within the study, barriers and facilitators for ACP implementation	Descriptive
Questionnaire	8	Characteristics	ACP coordinators	Baseline	Professional qualification and experience with counselling / ACP; motivation and expectations for the tasks, barriers and facilitators	descriptive

Table 1 Instruments for process evaluation: Implementation; Legend: ACP = advance care planning

Context

Data collection via	No.	Level	Target group	Data collection	Content	Analysis
Questionnaire	9	Ind./insti.	Nursing home staff	Baseline, t_2	Participants‘ characteristics, working experience, knowledge on advance directives, ACP, designated proxy, current practice, expectations regarding implementation of ACP in nursing homes	Descriptive, content analysis
Questionnaire	10	Ind.	Nursing home residents	Baseline, t_2	Attitude, motivation and preconditions to participate in ACP, knowledge on advance directives	Descriptive, content analysis
Questionnaire	11	Ind.	Resident´s relatives	Baseline, t_2	Delivery of ACP, proxy decision making, attitudes towards ADs and life-sustaining treatment	Descriptive, content analysis
Guideline- supported interview	23	Insti.	Nursing home management staff	Baseline, t_2	Coherence: knowledge, expectations; Cognitive participation: professional roles and common goals; ACP implementation in facility,	Qualitative content analysis
Guideline-supported Interview	27	Ind./insti./reg.	ACP-facilitators	t_2	Implementation of ACP, barriers and enabling factors	Qualitative content analysis
Group discussion	24	Ind./insti./reg.	ACP-coordinator	t_2	Implementation of ACP	Qualitative content analysis
Guideline-supported Interview	25	Reg.	Emergency staff, GP, others	Baseline	Attitudes towards ACP in general and in the local area, to active participation in a regional network, barriers and facilitating factors for regional implementation	Grounded theory
Questionnaire	14	Reg.	Emergency staff, GP, others	During intervention phase	Professional education, working experience, experience with ACP, AD guidelines, expectations, facilitators, barriers regarding ACP implementation	Descriptive, content analysis
Documentation	5	Regional national	Study staff	Continuous	Collection of documents: relevant social debates or political events with potential impact to the study	Content analysis

Table 2 Instruments for process evaluation: Context; Legend: ACP = advance care planning, Ind. = individual; Inst. = institutional; reg. = regional.
